# Combined Metabolic and Chemical (CoMetChem) Labeling Using Stable Isotopes—a Strategy to Reveal Site-Specific Histone Acetylation and Deacetylation Rates by LC–MS

**DOI:** 10.1021/acs.analchem.1c01359

**Published:** 2021-09-14

**Authors:** Alienke van Pijkeren, Jörn Dietze, Alejandro Sánchez Brotons, Anna-Sophia Egger, Tim Lijster, Andrei Barcaru, Madlen Hotze, Philipp Kobler, Frank J. Dekker, Peter Horvatovich, Barbro N. Melgert, Mathias Ziegler, Kathrin Thedieck, Ines Heiland, Rainer Bischoff, Marcel Kwiatkowski

**Affiliations:** † Institute of Biochemistry and Center for Molecular Biosciences Innsbruck, University of Innsbruck, Innsbruck 6020, Austria; ‡ Department of Analytical Biochemistry and Interfaculty Mass Spectrometry Center, Groningen Research Institute of Pharmacy, University of Groningen, Groningen 9700 AD, The Netherlands; § Department of Arctic and Marine Biology, UiT The Arctic University of Norway, Tromsø 9037, Norway; ∥ Chemical and Pharmaceutical Biology, Groningen Research Institute of Pharmacy, University of Groningen, Groningen 9700 AD, The Netherlands; ⊥ Department of Molecular Pharmacology, Groningen Research Institute for Pharmacy, University of Groningen, Groningen 9700 AD, The Netherlands; # Groningen Research Institute for Asthma and COPD, University Medical Center Groningen, University of Groningen, Groningen 9700 AD, The Netherlands; ¶ Department of Biomedicine, University of Bergen, Bergen 5009, Norway; ∇ Department of Pediatrics, Section Systems Medicine of Metabolism and Signaling, University of Groningen, University Medical Center Groningen, Groningen 9700 RB, The Netherlands; ○ Department for Neuroscience, School of Medicine and Health Sciences, Carl von Ossietzky University Oldenburg, Oldenburg 26129, Germany; ⧫ Neuro-SysMed, Department of Neurology, Haukeland University Hospital, Bergen, Norway, Department of Clinical Medicine, University of Bergen, Bergen 5021, Norway

## Abstract

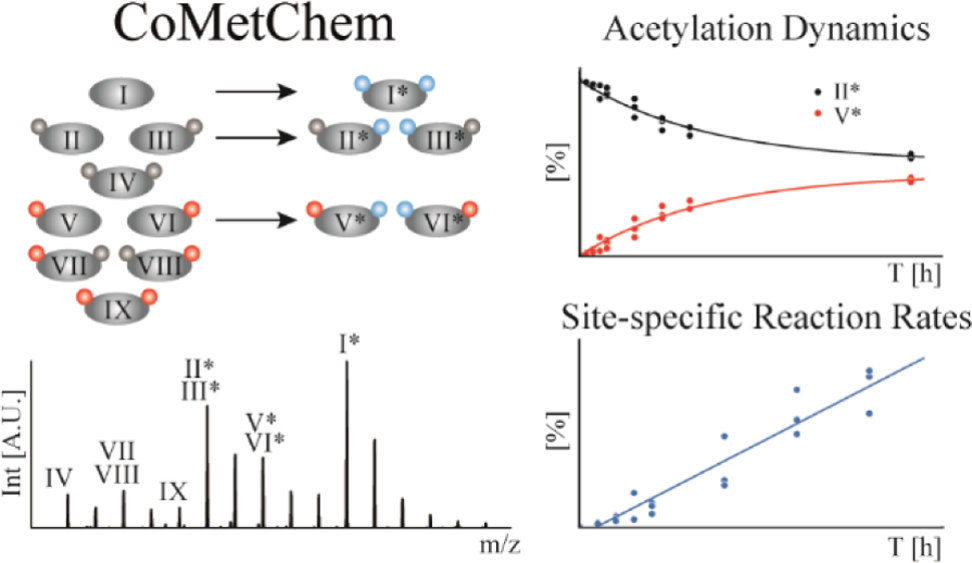

Histone acetylation is an important, reversible post-translational protein modification and a hallmark of epigenetic regulation. However, little is known about the dynamics of this process, due to the lack of analytical methods that can capture site-specific acetylation and deacetylation reactions. We present a new approach that combines metabolic and chemical labeling (CoMetChem) using uniformly 13C-labeled glucose and stable isotope-labeled acetic anhydride. Thereby, chemically equivalent, fully acetylated histone species are generated, enabling accurate relative quantification of site-specific lysine acetylation dynamics in tryptic peptides using high-resolution mass spectrometry. We show that CoMetChem enables site-specific quantification of the incorporation or loss of lysine acetylation over time, allowing the determination of reaction rates for acetylation and deacetylation. Thus, the CoMetChem methodology provides a comprehensive description of site-specific acetylation dynamics.

## Introduction

Living organisms are highly dynamic systems and constantly exposed to external changes and strive to maintain homeostasis. Post-translational modifications (PTMs) play a crucial role in this process, as they allow them to adapt to the protein function and activity in a rapid and highly dynamic manner. Histone lysine acetylation is a dynamic and reversible PTM regulated by histone acetyltransferases [HATs, also known as lysine acetyltransferases (KATs)] and histone deacetylases [HDACs, also known as lysine deacetylases (KDACs)].^1^ HATs regulate the transfer of acetyl groups from acetyl-coenzyme A (acetyl-CoA) to ε-amino groups of lysine residues, and HDACs catalyze the removal of acetyl groups from modified lysines. Histone acetylations are hallmarks of epigenetic regulation and are closely linked to the regulation of key cellular processes such as chromatin remodeling, transcriptional regulation, gene silencing, cell cycle progression, apoptosis, differentiation, DNA replication, DNA repair, and nuclear import.^1−5^ An imbalance in histone acetylation has been associated with a variety of human diseases including cancer,^6^ chronic inflammation,^7^ and neurological^8^ and metabolic disorders.^9^

Despite their central biological and medical importance, the dynamics and site specificity of acetylation events at histones are still poorly understood. Adjacent PTMs are generally accepted to influence one another,^10^ but the mechanisms and dynamics by which they control each other’s stability and turnover are largely unknown. An important reason for this gap in our knowledge is the lack of adequate methods for the site-specific analysis of acetylation dynamics and their interactions.

Histone modifications are still frequently detected using immunochemical techniques. However, site-specific antibodies against acetyllysines often lack specificity and cross-react with adjacent PTMs in the N-terminal histone tails.^11,12^ Moreover, while they allow to estimate the relative extent of a PTM under different conditions, immunochemical methods cannot be used to trace the turnover of PTMs. Mass spectrometry (MS) coupled to liquid chromatography (LC–MS) has emerged as a powerful method to investigate site-specific histone acetylation,^13^ providing reliable, site-specific identification and quantification of histone acetyllysines. MS-based methods also allow us to detect and quantify simultaneously occurring modifications within the same protein sequence in a single experiment. Nevertheless, quantitative analysis of histone acetylation is particularly challenging since the N-terminal histone tails are highly enriched in lysine and arginine residues in a short stretch of amino acids. Trypsin, the protease of choice for histone analysis by LC–MS in a bottom-up approach, does not cleave at the C-terminal site of acetylated lysines due to charge neutralization. Furthermore, the occurrence of adjacent lysines and arginines may lead to random missed cleavage events, resulting in a variety of short hydrophilic peptides, which are difficult to retain on reversed-phase (RP) columns. Thereby, a nonhomogeneous pool of peptide species is formed, in which the same acetylated residue can be present in different peptides. These chemically nonequivalent species complicate the accurate quantification of the different acetylated histone species due to their different retention times and ionization efficiencies.^14,15^ To generate a more homogenous pool of acetylated peptides and to increase the retention of hydrophilic peptides during RP chromatography, primary amino groups of extracted histones can be chemically propionylated or acetylated at the protein level prior to tryptic digestion.^16−18^ Smith et al. introduced chemical acetylation of unmodified lysine residues with deuterated acetic anhydride to quantify endogenous acetylation levels of the histone H4 protein.^16^ By derivatization with stable isotope-labeled acetic anhydride, the chemically acetylated lysines possessed the same ionization properties as the endogenously acetylated lysines. In another study, Feller et al. applied stable isotope-labeled acetic anhydride to investigate the effect of HATs and HDACs on combinatorial histone acetylation profiles.^19^ The chemical acetylation of unmodified lysines, using stable isotope-labeled acetylating reagents, allows us to quantify abundance levels of acetylated and nonacetylated histone species^20^ while retaining site-specific information.^21^

**Figure 1:**
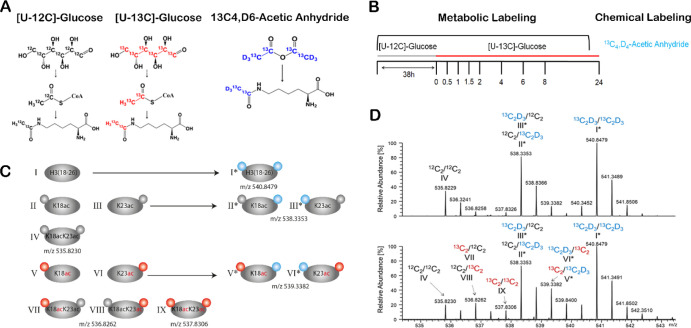
CoMetChem workflow for the analysis of site-specific histone acetylation dynamics. (A) CoMetChem combines metabolic and chemical labeling using stable isotopes. The metabolic labeling using [U-12C]-Glc or [U-13C]-Glc results in ^12^C_2_H_3_ (gray)- or ^13^C_2_H_3_ (red)-containing acetyllysines, respectively. The chemical acetylation of nonacetylated lysines using ^13^C_6_,D_6_-AA results in ^13^C_2_D_3_ (blue)-containing acetyllysines. (B) RAW264.7 cells were first cultured in a [U-12C]-glucose-containing medium (gray) followed by medium replacement to [U-13C]-glucose-containing medium (red). The nuclei were isolated from the samples at different time points, followed by nucleus isolation and histone extraction, chemical derivatization of unmodified lysine residues at the protein level using ^13^C_6_,D_6_-AA (blue), and tryptic digestion and quantitative LC–MS analysis. (C) Schematic representation of all possible acetylated H3 species covered by the H3(18-26) peptide generated metabolically (left panel, Roman numerals without asterisk) and the corresponding isotopologues generated by combination with chemical acetylation (right panel, Roman numerals with asterisk). Acetyl groups derived from [U-12C]-Glc are indicated in gray, [U-13C]-Glc-derived acetyl groups are indicated in red, and ^13^C_6_,D_6_-AA-derived acetyl groups are indicated by blue circles. (D) MS1 spectra of the H3(18-26) isotopologues upon CoMetChem labeling after medium exchange (*t* = 0) and 4 h of incubation with [U-13C]-Glc (*t* = 4 h). The spectra show the different H3(18-26) isotopologues, and the colors indicate whether the acetyl groups of the K18 (left) and K23 (right) residues are derived from [U-12C]-Glc (black), [U-13C]-Glc (red), or ^13^C_6_,D_6_-AA (blue).

These approaches allow us to detect the state of modification at specific residues, but they provide only a static snapshot of histone acetylation levels. They do not allow us to monitor site-specific acetylation dynamics, which is critical to follow the regulation, turnover, and interplay of histone modifications. Stable isotope-labeled metabolic precursors can be used to study the dynamics of proteins and PTMs.^22^ To study histone acetylation dynamics, Evertts et al.^23^ and Zheng et al.^24^ introduced metabolic labeling using uniformly 13C-labeled glucose ([U-13C]-Glc) as a metabolic precursor. [U-13C]-Glc is converted into 13C-labeled acetyl-CoA (Figure 1A), which serves as a substrate for the acetylation of lysine residues. MS analysis allows us to discriminate between newly 13C-labeled and pre-existing 12C-labeled acetylation sites, which enables determination of histone acetylation half-lives. However, half-lives of different acetylated species are not directly comparable. For example, an acetylated histone species whose substrate is 10 times more abundant compared to another one with the same half-life is turned over 10 times faster. Thus, to directly compare the dynamics of different acetylated species, substrate abundance must be considered. The substrate abundance of the species whose half-lives are to be compared can be determined by MS analysis. However, in order to be comparable, the analytes must have the same ionization efficiency, that is, the species to be compared must be chemically equivalent but distinguishable by MS. This is not the case for acetylated versus nonacetylated peptides.

To tackle this issue, we developed a combined metabolic and chemical labeling (CoMetChem) approach. CoMetChem combines [U-13C]-Glc-based metabolic labeling and chemical acetylation of nonacetylated lysine residues with ^13^C_4_,D_6_-acetic anhydride (^13^C_4_,D_6_-AA) at the protein level prior to LC–MS analysis. Thereby, all lysine residues of the histones are fully acetylated. Consequently, all peptide species generated during proteolytic digestion with identical amino acid sequences exhibit the same chemical properties, permitting accurate relative quantification by MS. Based on the mass shifts introduced by metabolic versus chemical labeling, CoMetChem allows us to quantify site-specific reaction rates of acetylation and deacetylation, a critical parameter to monitor the dynamics of PTMs and the effects of pharmacological modulators such as HDAC inhibitors.

## Experimental Section

### Chemicals

The chemicals used in this study are listed in the Supporting Information.

### Cell Culture and Metabolic Labeling with [U-13C]-Glucose

Experimental details of the cell culture conditions can be found in the Supplemental Experimental Section.

### Histone Extraction

For histone extraction, the cell nuclei were isolated first. The cells were collected and washed three times with PBS. For lysis, 500 μL of ice-cold nuclear isolation buffer [15 mM Tris–HCl, 15 mM NaCl, 60 mM KCl, 5 mM MgCl_2_, 1 mM CaCl_2_, 250 mM sucrose, 1 mM dithiothreitol (DTT), 1 mM phenylmethylsulfonyl fluoride, 10 mM nicotinamide, 10 mM sodium butyrate, and 0.01% sodium deoxycholate (SDC)] was used. The homogenized cells were incubated for 10 min on ice and washed three times with the nuclear isolation buffer without SDC. Histones were extracted from the nuclei using 0.2 M HCl. For more details, see the Supporting Information.

### Chemical Acetylation Using ^13^C_4_,D_6_-AA and Tryptic Digestion

Sodium borate (SB), ammonium bicarbonate, and triethylammonium bicarbonate (TEAB) were used as reaction buffers to study the chemical acetylation efficiency at the protein level. The experimental details for the comparison of the reaction buffers can be found in the supplemental experimental section. For CoMetChem, 6 μg of the histone extract was diluted in HPLC-H_2_O to a final volume of 50 μL. For reduction, the samples were incubated with 10 mM DTT and dissolved in 100 mM SB (pH 8.5) for 10 min at 57 °C on a shaker (600 rpm). Afterward, the samples were alkylated with 20 mM iodoacetamide (IAA), dissolved in 100 mM SB, and incubated for 30 min at room temperature in the dark. Subsequently, free IAA was quenched by adding 10 mM DTT (dissolved in 100 mM SB). Subsequently, 50 μL of 100 mM SB (pH 8.5) was added. Then, 1 μL of 4.1 mM ^13^C_4_,D_6_-AA (dissolved in water-free dimethyl sulfoxide) was added, followed by incubation on a thermomixer (1000 rpm) at 25 °C for 10 min. The chemical acetylation was repeated twice. O-Acetylation was reverted by adding hydroxylamine to a final concentration of 7.6 mM, followed by incubation on a thermomixer (450 rpm) at 25 °C for 120 min. For tryptic digestion, the samples were incubated with 1 μL of trypsin solution (*c* = 0.2 μg/μL sequencing grade modified trypsin, dissolved in trypsin resuspension buffer, Promega, Walldorf, Germany) for 16 h at 37 °C. Afterward, the samples were acidified by adding formic acid (FA) to a final concentration of 0.1% FA.

### LC–MS/MS Analysis

For LC–MS/MS analysis, 100 ng of the tryptic peptide digests was injected on a nano-ultrapressure LC system (Dionex UltiMate 3000 RSLCnano pro flow, Thermo Scientific, Bremen, Germany). The nano-UPLC system was coupled via an electrospray ionization (ESI) source to a QExactive Plus (Thermo Scientific, Bremen, Germany) or a tribrid orbitrap mass spectrometer (Orbitrap Fusion Lumos, Thermo Scientific, San Jose, CA, USA) to study the chemical acetylation efficiency of extracted histones and to analyze CoMetChem-derived isotopologue species. All details of the RP separation and mass spectrometric parameters can be found in the Supporting Information. For the analysis of CoMetChem-derived isotopologue species, peptides were separated with a linear gradient from 5 to 37.5% B in 25 min, followed by 37.5–62.5% B in 5 min (B: 80% ACN, 0.1% FA in HPLC-H_2_O; A: 0.1% FA in HPLC-H_2_O). MS analysis was carried out in the data-dependent acquisition mode. Scan mode 1: MS/MS spectra were acquired in the top-speed mode. Scan mode 2: for isobaric H3(18-26) isotopologues, an inclusion list with a defined *m*/*z* (±5 ppm), charge state, and retention time window was used (*m*/*z* 536.8228, *z* = 2; *m*/*z* 538.3322, *z* = 2; *m*/*z* 539.3356, *z* = 2, and RT: 22.07–23.07 min). MS/MS spectra were recorded in the orbitrap with a resolution of 7500 FWHM at *m*/*z* 200 (scan mode 1: maximum injection time = 50 ms, AGC target = 100%, intensity threshold: 2.5 × 10^4^ quadrupole isolation width: 0.4 Da, scan mode 2: maximum injection time = 100 ms, AGC target = 100%, intensity threshold: 5 × 10^5^, and quadrupole isolation width: 0.4 Da).

### LC–MS/MS Data Processing

Experimental details of the bioinformatics data analysis of the chemically acetylated histone species can be found in the Supporting Information. For ComMetChem, data analysis was performed in FreeStyle 1.6 (Version 1.6.75.20, Thermo Scientific, Bremen, Germany). For the different H3(18-26) and H4(4-17) isotopologues, extracted ion chromatograms (EICs) of all isotopes of the isotopic distributions were generated with a mass tolerance of ±3 ppm. For peak detection, the Genesis algorithm was used with the following parameters: percent of the highest peak: 10, minimum peak height (signal/noise): 5, signal-to-noise threshold: 3, and tailing factor: 3. The peak area and the peak height were exported for each isotope of the isotopic distributions of the different isotopologue species to *.csv. For natural isotope abundance correction, the python package PICor was used, which uses a theoretical correction approach based on statistical distributions of each isotopologue. The detailed approach is described on bioRxiv,^25^ and the source code is available under https://github.com/MolecularBioinformatics/PICor. To determine site-specific acetylation levels for the MS1 isobaric H3(18-26) isotopologue species and single-acetylated H4(4-17) isotopologue species, the relative abundances of site-specific acetyllysine-containing fragment ions were quantified using Pipelines and Systems for Threshold-Avoiding Quantification of LC–MS/MS data (PASTAQ).^26^ The details are described in the Supplemental Experimental Section and the Supplemental Tutorial.

### Half-Life and Abundance-Corrected Turnover Calculation

The half-lives of the individual acetylation sites of the single- and double-acetylated H3(18-26) species were calculated based on fitted exponential growth functions (see eq 1) to the measured fraction of the heavy (H) to light (L) isotopologue ratios over time (*t*). The SciPy^27^ optimization function curve fitting was used for determining the pre-factor *p* and the exponential factor *k* along with their respective standard deviations σ_*p*_ and σ_*k*_; see eqs 2 and 3. In the case of the double-acetylated species, we fitted an exponential decay function with the *y*-axis intercept set to 100%, to the ratio of the double-acetylated species divided by the sum of the single and double species; see eq 4
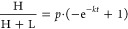
1
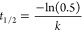
2
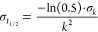
3
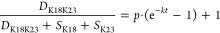
4

The abundance-corrected turnover was calculated by multiplying the turnover rate (*k*) with the abundance (*A*) of the respective peptide species. The half-lives and abundance-corrected turnovers are listed in Table S1.

### Calculation of Acetylation and Deacetylation Rates

The SciPy linregress function was used to derive the abundance A of the respective substrates and the initial slope, which was used to calculate the net acetylation and deacetylation rates υ of the peptide species according to eqs 5 and 6. It should be noted that the substrate for the deacetylation reaction (eq 5) is the abundance of the same peptide species *x* at time 0, whereas for the acetylation reaction (eq 6), the substrate is the corresponding nonacetylated species
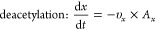
5
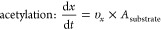
6

The acetylation and deacetylation rates are listed in Table S2. The graphs were plotted with Python 3.8 using the Python libraries matplotlib and seaborn.

## Results and Discussion

### Chemical Acetylation

Unmodified lysine residues are chemically acetylated using stable isotope-labeled ^13^C_4_,D_6_-AA, which generates ^13^C_2_D_3_-containing acetyllysines (Figure 1A). These acetyl groups have a mass increment of 5.0252 and 3.0185 Da compared to those derived from [U-12C]-Glc (^12^C_2_H_3_) and [U-13C]-Glc (^13^C_2_H_3_), respectively. The mass increments can be resolved in state-of-the art TOF and orbitrap mass analyzers, and the different isotopologues have the same ESI efficiencies. Complete chemical acetylation of the nonacetylated lysine residues at the protein level is essential for the CoMetChem strategy to ensure that only chemically equivalent species arise from the tryptic digestion of a given protein. We obtained an almost complete derivatization with both TEAB (99.7 ± 1.0%, *c* = 100 mM, pH 8.5) and SB (99.4 ± 1.6%, *c* = 100 mM, pH 8.5) as reaction buffers (Figure S1A). The derivatization efficiency was evaluated by LC–MS analysis of 14 histone lysine residues including the H3(18-26) and H4(4-17) peptides harboring two and four lysine residues, respectively. Acetylating lysine residues that are in close proximity to each other was particularly challenging. While both TEAB (99.9 ± 0.2%) and SB (99.8 ± 0.2%) resulted in a near-complete acetylation for the H3(18-26) peptide, which contains two lysine residues (Figure S1B), SB (97.9 ± 0.3%) resulted in a significantly higher acetylation efficiency for the four-lysine-containing H4(4-17) peptide compared to TEAB (96 ± 0.5%) (Figure S1C). Thus, we continued to use SB as a reaction buffer for chemical acetylation with ^13^C_4_,D_6_-AA.

### Site-Specific Acetylation Turnover

To analyze site-specific acetylation turnover by CoMetChem in cell culture, we combined metabolic and chemical labeling with [U-13C]-Glc and ^13^C_4_,D_6_-AA, respectively. The macrophage cell line RAW 264.7 was first cultured in [U-12C]-Glc-containing medium and then switched to [U-13C]-Glc-containing medium (*t* = 0) (Figure 1B). Histones were isolated at different time points after *t* = 0 over a period of 24 h and subjected to chemical derivatization with ^13^C_4_,D_6_-AA, trypsin digestion, and LC–MS/MS-analysis. H3(18-26) peptide species, generated by tryptic digestion from H3 protein and covering a region with two adjacent acetylation sites (K18 and K23), were chosen as an example. Combined metabolic and chemical labeling by CoMetChem results in nine different H3(18-26) peptide isotopologues (Figure 1C). At the MS1 level, only six of these species could be resolved (Figure 1D), as the remaining species are isobaric and can only be distinguished based on their fragment ion spectra (Figure S2). At *t* = 0, before the addition of [U-13C]-Glc, the following isotopologues can be detected (Figure 1C,D):

I: The nonacetylated H3(18-26) peptide which contains two ^13^C_4_,D_6_-AA-derived acetyl groups (*m*/*z* 540.8479).

II, III: The endogenously single-acetylated H3(18-26) species, which contain a [U-12C]-Glc-derived acetyl group at K18 (II) or K23 (III) and a ^13^C_4_,D_6_-AA-derived acetyl group at K23 (II)and K18 (III), respectively. Both species are isobaric (*m*/*z* of 538.3353) and cannot be distinguished at MS1.

IV: The H3(18-26) peptide with two endogenous [U-12C]-Glc-derived acetyl groups (*m*/*z* 535.8230).

Cultivation in the presence of [U-13C]-Glc in combination with chemical acetylation using ^13^C_4_,D_6_-AA led to the formation of the following additional H3(18-26) isotopologues (Figure 1C,D):

V, VI: The endogenously single-acetylated H3(18-26) species which contain a [U-13C]-Glc-derived acetyl group at K18 (V) or K23 (VI) and a ^13^C_4_,D_6_-AA-derived acetyl group at K23 (V) and K18 (VI), respectively. Both species are isobaric (*m*/*z* 539.3382) and cannot be distinguished at MS1.

VII, VIII: The endogenously double-acetylated H3(18-26) species which contain a [U-13C]-Glc-derived acetyl group at K18 (VII) or K23 (VIII) and a [U-12C]-Glc-derived acetyl group at K23 (VII) and K18 (VIII), respectively, which are isobaric at MS1 (*m*/*z* 536.8262).

IX: The endogenously double-acetylated H3(18-26) species which contains two [U-13]-Glc-derived acetyl groups (*m*/*z* 537.8306).

The single-acetylated species I and II along with V and VI, and the metabolically double-acetylated species VII and VIII are positional isomers and isobaric at MS1 (Figure 1D) but are distinguishable based on their fragment ion spectra (Figure S2B). In the case of the K18ac and K23ac positional isomers derived from [U-13C]-Glc (V, VI), the b3 fragment ions contained either an acetyllysine derived from [U-13C]-Glc for K18 (*m*/*z* 414.2593) or a chemically acetylated lysine for K23 (*m*/*z* 417.2828) (Figure S2C). The y7 fragment ions contained either a chemically acetylated lysine for K18 (*m*/*z* 777.4940) or an acetyllysine derived from [U-13C]-Glc for K23 (*m*/*z* 774.4755, Figure S2D).

As the fragment ions differ in their stable isotopic composition and their *m*/*z* values, the stoichiometry of the K18ac and K23ac species can be determined based on the relative abundance of the respective fragment ions.

Since chemical derivatization leads to chemically equivalent isotopologues, it enables quantification of the relative abundance of the different acetylated H3(18-26) peptides. However, another factor contributes to the complexity of the isotopologue mixture, namely, the naturally occurring 13C in the peptide backbone resulting in additional overlapping isotopologues at the MS1 level (Figure 1D). We therefore included an isotope correction in the data analysis to ensure accurate quantification. The Python-based isotope correction pipeline PICor used a skewed matrix algorithm, which corrects each different isotopologue species with its own set of theoretical correction factors.^25^ A detailed tutorial on bioinformatics workflow and data processing can be found in the Supporting Information.

**Figure 2:**
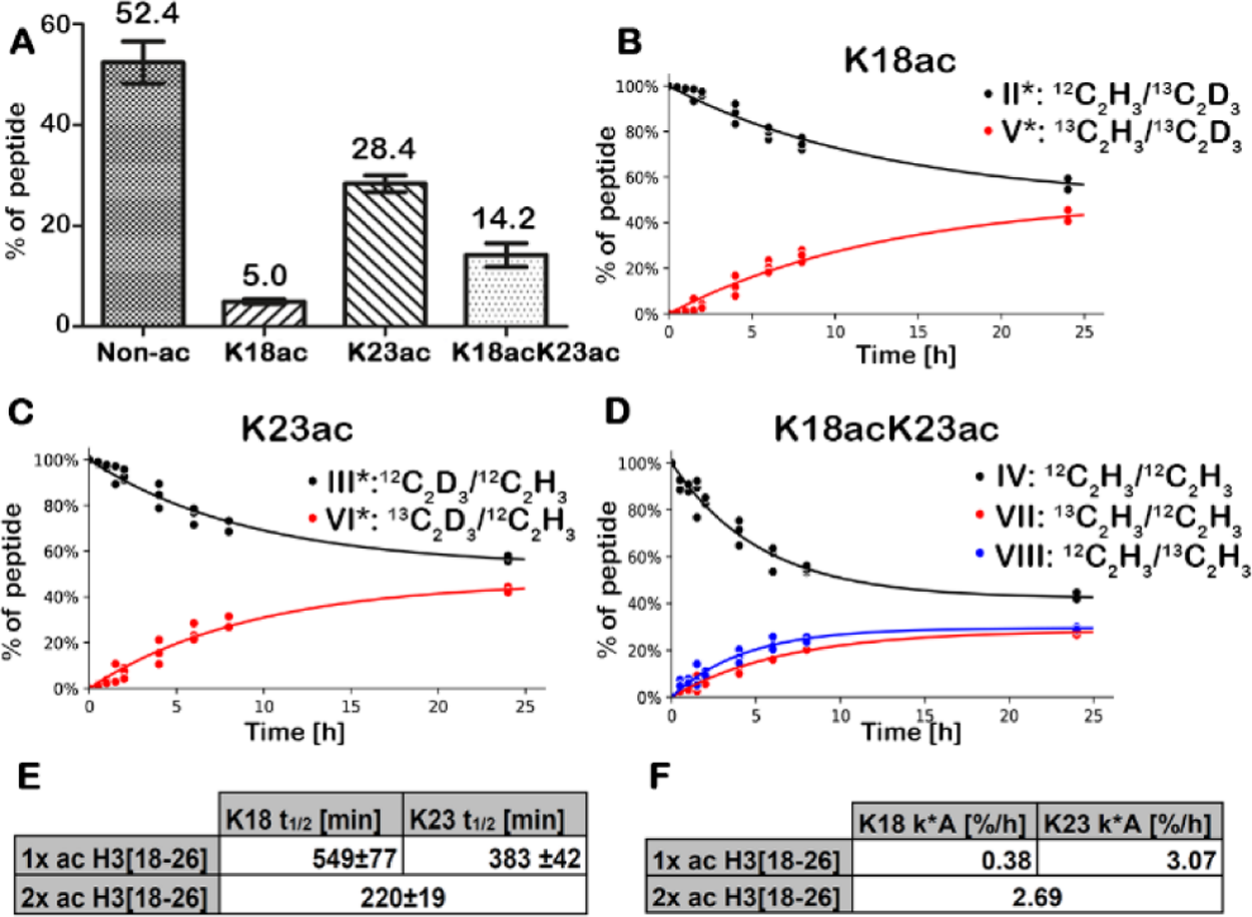
Site-specific abundance levels, half-lives, and turnovers of the single- and double-acetylated H3(18-26) peptide species. (A) Bar charts (median with standard deviation) showing abundance levels of the nonacetylated (Non-ac), single-acetylated (K18ac and K23ac), and double-acetylated (K18acK23ac) H3(18-26) species. (B,C) Site-specific label incorporation and label loss of the single-acetylated H3(18-26) species with an acetyllysine at K18ac (B) or K23ac (C). (D) Site-specific label incorporation and label loss of K18ac and K23ac of the double-acetylated H3(18-26) species. (E) Site-specific half-lives of K18ac and K23ac of the single-acetylated (1× ac) and half-life of the double-acetylated (2× ac) H3(18-26) species. (F) Site-specific turnover of K18ac and K23ac of single-acetylated (1×) ac and turnover of the double-acetylated (2× ac) H3(18-26) species. *n* = 3 independent experiments.

Comparison of the relative abundance levels of the different acetylated H3(18-26) species revealed that the endogenously nonacetylated species was the most abundant (52.4%), followed by the single-acetylated K23ac species (28.4%), the double-acetylated K18acK23ac species (14.2%), and the single-acetylated K18ac species (5.0%) (Figure 2A). The levels were stable over the time course of 24 h and across all biological replicates (Figure S3), highlighting the tight control of the acetylation levels at these residues.

To monitor acetylation dynamics, site-specific label incorporation for the single- and double-acetylated species was quantified. We observed a maximum relative label exchange of 40% for all acetylated species (Figure 2B–D), which is comparable to the results reported by Zheng et al.,^24^ where [U-13C]-Glc was used for metabolic labeling without additional chemical acetylation. Individual turnover rates (*k*) were calculated by fitting experimental values to exponential growth or decay functions to calculate the site-specific half-lives (*t*_1/2_ = ln 2/*k*) (Figure 2E). The single-acetylated species K23ac showed a half-life of 383 ± 42 min, whereas the acetyllysine at K18 showed a half-life of 549 ± 77 min. The double-acetylated H3(18-26) peptide (Figure 1C, IV) can be converted into three different isotopologues (Figure 1C, VII, VIII, and IX). Thus, the exponential growth or decay functions used above to determine half-lives and turnover rates can only be used to calculate the total half-life of the double-acetylated peptide and cannot be used to correctly calculate site-specific turnover. The double-acetylated species showed a half-life of 220 ± 19 min.

As stated earlier, half-lives are not directly comparable for substrates with different abundances. Using the CoMetChem approach, which generates chemically equivalent species, we were, however, able to calculate the individual acetylation turnover for the single-acetylated and double-acetylated H3(18-26) species. For this, the turnover rate (*k*) was multiplied with the abundance (*A*) of the respective peptide species. The single-acetylated K23ac and K18ac species showed a turnover of 3.07%/h and 0.38%/h (Figure 2F), respectively. Thus, the single-acetylated species had an eight times faster turnover at K23ac compared to K18ac. The overall turnover of the double-acetylated peptide was calculated to be 2.69%/h.

### Site-Specific Reaction Rates for Acetylation and Deacetylation

**Figure 3:**
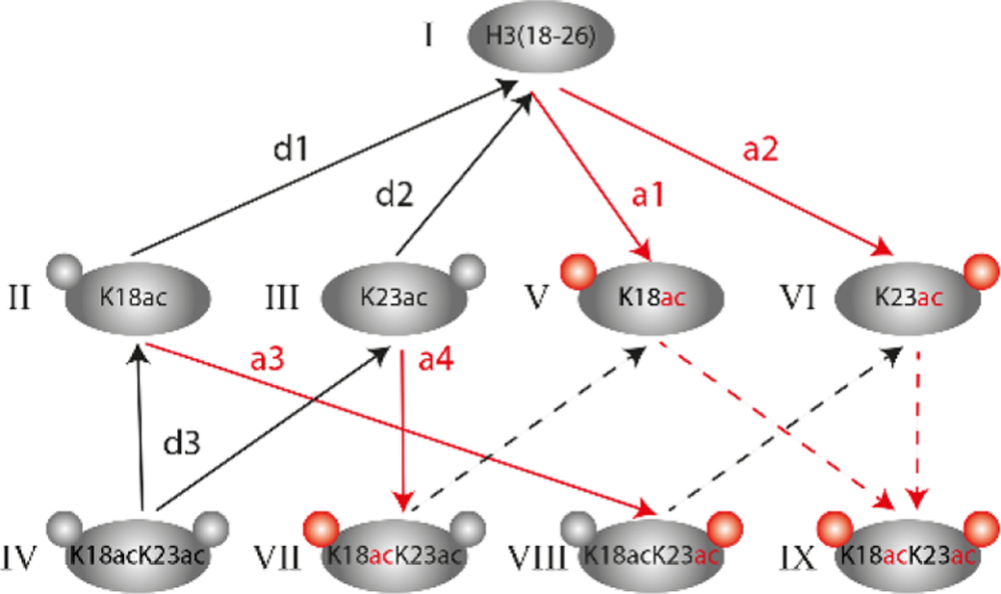
Schematic representation of possible in vivo generated H3[18-26] species and reactions occurring in the metabolic labeling approach. Red arrows represent acetylation reactions (marked as “a”), and black arrows represent deacetylation reactions (marked as “d”). Dashed arrows represent reactions for which initial flux fitting is not possible as the respective labeled substrates are not present at time point *t* = 0.

The calculation of turnover does still represent a simplified description of the acetylation dynamics, as it does not provide a comprehensive description of all modification events that occur. Each acetylation turnover event involves at least one acetylation and one deacetylation reaction. For example, the nine different H3 species covered by the H3(18-26) peptide are interconverted by at least 12 different reactions (Figure 3).

Since CoMetChem enables the direct comparison of the abundance of all peptide species, we were able to determine individual reaction rates of acetylation and deacetylation for all H3(18-26) species. However, it is not possible to calculate rates for species that are not present before label addition (dashed arrows in Figure 3). For our calculations, we adapted an approach presented earlier by Zheng et al.^28^ for histone methylation analysis. The strategy is based on linear fitting of the initial interconversion fluxes, where the fluxes are the slopes of the time courses and thus represent the increase or decrease in the different acetylated peptide species over time (Figure S4A–G). This approach is based on the simplified assumption that the substrate concentration is initially relatively constant, and therefore, the corresponding products initially increase linearly with time. To estimate the acetylation and deacetylation rates (υ), the measured fluxes need to be divided by the relative abundance of the respective substrate at *t* = 0. We determined site-specific reaction rates of acetylation (Figure S4A,B) and deacetylation (Figure S4C,D) for the single-acetylated peptide. The acetylation rate at K23 (a2, Figure S4B) was determined to be 0.019 ± 0.002/h, whereas the deacetylation rate at the same site (d2, Figure S4D) was 0.059 ± 0.006/h. For K18, we obtained an acetylation rate (a1, Figure S4A) of 0.0036 ± 0.0002/h and a deacetylation rate (d1, Figure S4C) of 0.032 ± 0.008/h. The comparison of the single-acetylated H3(18-26) species revealed that the acetylation rate for K23 (a2) is approximately five times higher than the acetylation rate at K18 (a1). The difference in site-specific deacetylation rates at K18 (d1) and K23 (d2) is not as pronounced as in the acetylation rates, but K23ac still showed a two times faster deacetylation rate compared to K18ac. Thus, acetylation and deacetylation rates can substantially differ between lysines within the same peptide.

As we cannot determine which of the [U-12C]-Glc-derived single-acetylated species (II or III, Figure 3) is produced when the [U-12C]-Glc-derived double-acetylated species (IV, Figure 3) is deacetylated, it was not possible to deduce the site-specific turnover for double-acetylated species. We can, however, deduce the total deacetylation rate for double-acetylated species (represented by reaction d3 in Figure 3). With 0.084/h±0.027 (d3, Figure S4G), it was approximately equal to the sum of the rates (d1 and d2) determined for the site-specific deacetylation rates of the single-acetylated species (Figure S4C,D). This indicates that in the H3(18-23) peptide, the site-specific deacetylation rates were not affected by the acetylation state of the nearby lysine residue.

In contrast to deacetylation rates, the CoMetChem approach allows us to determine site-specific acetylation rates for the formation of the double-acetylated peptide. This is based on the conversion of the single-acetylated H3(18-26) species II and III into the double-acetylated species VIII and VII by reactions a3 and a4, respectively (Figure 3). The site-specific acetylation rates at K18 (a4) and K23 (a3) for the double-acetylated species were calculated to be 0.008 ± 0.001/h and 0.054 ± 0.006/h, respectively (Figure S4E,F). They are thus two–three times faster than for the single-acetylated peptides. This indicates that an already existing acetylation promotes the acetylation of the nearby site for the H3(18-23) species.

In addition, we determined site-specific reaction rates for the single-acetylated H4(4-17) species using CoMetChem. For this, we first quantified the overall abundance level of the nonacetylated (43.5%), single- (27.8%), double-acetylated (16.1%), and three (6.2%) and four times (6.4%) acetylated H4(4-17) species (Figure S5A). We then quantified site-specific abundance levels for the single-acetylated H4(4-17) species K5ac, K8ac, K12ac, and K16ac to determine site-specific reaction rates for deacetylation and acetylation. The K16ac species showed, with 22.9%, the highest abundance of the single-acetylated H4(4-17) species (Figure S5B). In contrast, very low abundances were observed for K5ac (1%), K8ac (1.5%), and K12ac (2.4%). Thus, site-specific reaction rates were considered for the single-acetylated K16ac species only. The single-acetylated H4(4-17)-K16 species showed a deacetylation rate (d4) of 0.048 ± 0.005/h at K16 and an acetylation rate (a4) of 0.015 ± 0.001/h (Figure S6). These results demonstrate that CoMetChem is applicable to determine the reaction rates of acetylation and deacetylation of different acetylated histone species. In this study, site-specific reaction rates of acetylation and deacetylation were determined for single-acetylated H4(4-17) species, as all isobaric isotopologue species could be distinguished at the MS2 level. To determine site-specific reaction rates for the double-acetylated and three and four times acetylated H4(4-17) species, MSn experiments are required to distinguish isobaric species.

### Site-Specific Reaction Rates for Acetylation and Deacetylation after Treatment with HDAC Inhibitors

We further explored the CoMetChem approach to investigate site-specific acetylation dynamics upon pharmacological intervention with two HDAC inhibitors. We used suberanilohydroxamic acid (SAHA)^29^ (Figure S7A), which inhibits class I, II, and IV HDACs, and the benzamide derivative entinostat (MS-275)^30^ (Figure S7B), which inhibits the class I HDACs HDAC1 and HDAC3. RAW264.7 cells were first cultured in [U-12C]-Glc-containing medium for 22 h, followed by a pre-incubation of 16 h with either SAHA or MS-275 (Figure S7C). Afterward, the culture medium was replaced with [U-13C]-Glc-containing medium including the corresponding HDAC inhibitor.

As expected, the HDAC inhibitors led to decreased deacetylation. For H4(4-17), this is reflected in the significant decrease in nonacetylated species from 43.5% (control, C) to 18.0% (MS-275, M) and 12.8% (SAHA, S) and a significant increase in the two (C: 16.1%, M: 21.9%, S: 22.0%), three (C: 6.2%, M: 14.7%, S: 17.3%), and four (C: 6.4%, M: 21.5%, S: 26.3%) times acetylated H4(4-17) peptide (Figure S5A). For the single-acetylated K16ac H4(4-17) species, treatment with MS-275 (20.1%) and SAHA (18.4%) resulted in significantly lower acetylation levels compared to the control (22.9%) (Figure S5B). Although HDACi treatment did not result in higher acetylation levels for the single-acetylated H4(4-17) species, analysis of site-specific reaction rates revealed that treatment with MS-275 and SAHA resulted in complete inhibition of deacetylation reactions at K16 (d4) compared to the control (Figure S6B) and to decreased acetylation rates at the same site (Figure S6C).

**Figure 4:**
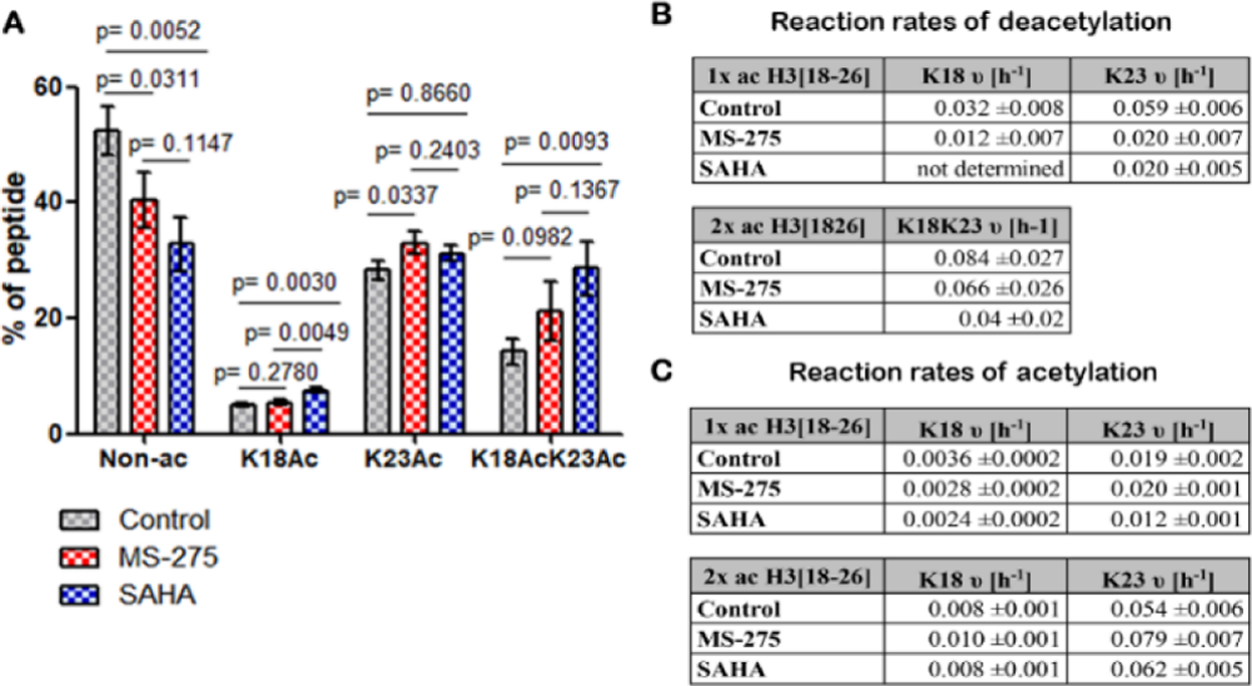
Effect of the histone deacetylase inhibitors MS-275 and SAHA on the site-specific acetylation levels and the acetylation and deacetylation rates of the single-acetylated and double-acetylated H3(18-26) peptide species. (A) Bar chart (mean with standard deviation) showing abundance levels of the nonacetylated (Non-Ac), single-acetylated (K18ac and K23ac), and double-acetylated (K18acK23ac) H3(18-26) species after 16 h of incubation with MS-275, SAHA, or the carrier (control). (B) Site-specific deacetylation rates of K18ac and K23ac of the single-acetylated (1× ac) H3(18-26) species and deacetylation rate for the double-acetylated peptide. (C) Site-specific acetylation rates of K18ac and K23ac of the single-acetylated (1× ac) and double-acetylated (2× ac) H3(18-26) species. *n* = 3 independent experiments. Statistical analyses were performed using the two-tailed unpaired *t*-test.

Next, we investigated the effect of the HDACi on the H3(18-26) species. As expected, HDAC inhibitors also resulted in decreased deacetylation. This is reflected in the significant increase in the double-acetylated H3(18-26) from 14.2 to 21.1% with MS-275 and to 28.6% with SAHA (Figure 4A). In addition, the abundance of the corresponding endogenously nonacetylated H3(18-26) species was reduced from 52.4 to 40.5% with MS-275 and to 32.8% with SAHA. The changes in the single-acetylated peptides were not pronounced. Still, the abundance of the single-acetylated K18ac species was significantly increased upon SAHA treatment, while MS-275 did not alter the abundance of this site. In contrast, the abundance of the single-acetylated K23ac species was elevated by MS-275 but not upon SAHA exposure. These results point to differences in site specificity between HDACs targeted by MS-275 or SAHA.

Finally, we evaluated the effects of SAHA and MS-275 on the rates of acetylation and deacetylation of the single- and double-acetylated H3(18-26) species (Figures 4 and S8). The deacetylation rate for the double-acetylated H3(18-26) species (d3) was 0.066 ± 0.026/h with MS-275 and thus similar to the control (Figures 4B and S9). In contrast, SAHA decreased the deacetylation rate from 0.084 ± 0.027/h to 0.04 ± 0.02/h (Figures 4B and S10). A comparison of the site-specific deacetylation rates for the single-acetylated H3(18-26) species revealed that MS-275 reduced the deacetylation rates at K23(d2) from 0.059 ± 0.006/h to 0.020 ± 0.007/h and from 0.032 ± 0.008/h to 0.012 ± 0.007/h at K18(d1). With SAHA, the acetylation at K18 appeared to be relatively constant, indicating a near-complete inhibition of the deacetylation at this site. We note, however, that this requires further investigation, as the measured values were at the detection limits of the method. The deacetylation rate at K23 was reduced from 0.059 ± 0.006/h to 0.020 ± 0.005/h upon exposure to SAHA (Figure 4B). Treatment with SAHA or MS-275 slightly decreased the acetylation rates at K18 for the single-acetylated (a1) H3(18-26) species (Figure 4C). While SAHA showed no effect at K18 for the double-acetylated H3(18-26) species, treatment with MS-275 resulted in an increase in acetylation rates at this site. For K23, treatment with MS-275 resulted in acetylation rates similar to the control for the single-acetylated (a2) H3(18-26) species, whereas treatment with SAHA resulted in decreased acetylation rates. In contrast, treatment with MS-275 resulted in higher acetylation rates at K23 for the double-acetylated (a3) species compared to the control, while SAHA and the control showed similar acetylation rates.

Taken together, these results demonstrate that our CoMetChem approach provides a comprehensive description of the dynamics of reversible lysine acetylation by determining the site-specific reaction rates of deacetylation and acetylation. Using CoMetChem, we were able to describe the dynamic effects of HDACs in a site-specific manner using HDAC inhibitors.

## Conclusions

With the CoMetChem methodology, we present a novel approach for the analysis of site-specific histone acetylation dynamics and reaction rates. Compared to the combinatorial use of metabolic [U-13C]-Glc labeling and chemical acetylation with propionic anhydride, the combinatorial use of stable isotope-labeled metabolic precursors ([U-13C]-Glc) and chemical acetylation using ^13^C_4_,D_6_-AA results in fully acetylated tryptic peptide isotopologues, which are chemically equivalent. Thus, the isotopologues only differ in their mass due to their isotopic composition while having the same ionization and ion transmission efficiencies during MS analysis. Due to the chemical equivalence of the peptide species, site-specific abundance levels can be determined with high precision, and site-specific reaction rates of acetylation and deacetylation can be quantified. These reaction rates describe the acetylation dynamics much more accurately than half-lives and allow the kinetics of individual acetylation sites to be directly compared. Thereby, CoMetChem goes beyond half-lives and turnovers, as site-specific reaction rates of acetylation and deacetylation can be determined, allowing a comprehensive assessment of the dynamics of this reversible modification. CoMetChem expands the repertoire of dynamic proteomics methods and represents a particularly valuable approach for deciphering the site selectivity of HDACs and protein acetyl transferases, facilitating the development of HDAC inhibitors with well-characterized substrate profiles.

## Supporting Information Available

MS1 information of H3(18-26) isotopologues (XLSX)
MS2 information of H3(18-26) isotopologues (XLSX)
MS1 information of H4(4-17) isotopologues (XLSX)
MS2 information of H4(4-17) isotopologues (XLSX)
Comparison of the derivatization efficiency of intact histones, identification and quantification of H3[18-26] positional isomers, site-specific abundance of the single- and double-acetylated H3(18-26) peptide species, initial flux analysis to determine reaction rates of acetylation and deacetylation, effect of the histone deacetylase inhibitors MS-275 and SAHA on the H4(4-17) acetylation level, chemical structure of the HDAC inhibitors and the workflow for the metabolic labeling, site-specific label incorporation for K18ac and K23ac of the single- and double-acetylated H3(18-26) species, flowchart showing the different steps of the CoMetChem data analysis pipeline, MS1 spectrum of the H3(18-26) isotopologues, extracted ion chromatograms (EICs) of the H3(18-26) isotopologue species, abundances of H3(18-26) isotopologues before and after PICor correction, graphical user interface (GUI) of PASTAQ, gaussian fitting of detected y7-ion fragments, site-specific label incorporation and label loss of the single-acetylated H3(18-26), linear fit over the abundances of the single-12C acetylated species, calculated half-lives of acetylated H3(18-26) species, calculated reaction rates of acetylation and deacetylation of the H3(18-26) species, calculated reaction rates of acetylation and deacetylation of the single-acetylated H4(4-17) species, intensities of the H3(18-26) isotopologues, input for PICor correction, sample table for MS2 fragment ion quantification, fragment table fragment ion quantification, and fragment ion quantification results (PDF)
