# Supplementary material for: Combined Metabolic and Chemical (CoMetChem) Labeling Using Stable Isotopes—a Strategy to Reveal Site-Specific Histone Acetylation and Deacetylation Rates by LC–MS
Source: Anal Chem. 2021 Sep 14;93(38):12872–80. doi: 10.1021/acs.analchem.1c01359 (PMC8482368; doi:10.1021/acs.analchem.1c01359)
Supplement: Supplementary file 5 — ac1c01359_si_005.pdf [file ac1c01359_si_005.pdf]

Supporting Information

Combined Metabolic and Chemical (CoMetChem) labeling using stable isotopes – A strategy to reveal site-specific histone acetylation and deacetylation rates by LC-MS.

Alienke van Pijkeren [1, 2‡], Jörn Dietze [3‡], Alejandro Sánchez Brotons [2‡], Anna-Sophia Egger [1‡], Tim Lijster [2], Andrei Barcaru [2], Madlen Hotze [1], Philipp Kobler [1], Frank J. Dekker [4], Peter Horvatovich [2], Barbro N. Melgert [5, 6], Mathias Ziegler [7], Kathrin Thedieck [1, 8, 9*], Ines Heiland [3, 10*], Rainer Bischoff [2*], Marcel Kwiatkowski [1, 2, 5, 6*]

1 Institute of Biochemistry and Center for Molecular Biosciences Innsbruck, University of Innsbruck, Innsbruck, 6020 Innsbruck, Austria

2 Department of Analytical Biochemistry and Interfaculty Mass Spectrometry Center, Groningen Research Institute of Pharmacy, University of Groningen, Groningen, 9700 AD Groningen, The Netherlands

3 Department of Arctic and Marine Biology, UiT The Arctic University of Norway, Tromsø, 9037 Tromsø, Norway

4 Chemical and Pharmaceutical Biology, Groningen Research Institute of Pharmacy, University of Groningen, Groningen, 9700 AD Groningen, The Netherlands

5 Department of Molecular Pharmacology, Groningen Research Institute for Pharmacy, University of Groningen, Groningen, 9700 AD Groningen, The Netherlands

6 Groningen Research Institute for Asthma and COPD, University Medical Center Groningen, University of Groningen, Groningen, 9700 AD Groningen, The Netherlands

7 Department of Biomedicine, University of Bergen, Bergen, 5009 Bergen, Norway

8 Department of Pediatrics, Section Systems Medicine of Metabolism and Signaling, University of Groningen, University Medical Center Groningen, Groningen, 9700 RB Groningen, The Netherlands

9 Department for Neuroscience, School of Medicine and Health Sciences, Carl von Ossietzky University Oldenburg, Oldenburg, 26129 Oldenburg, Germany

10 Neuro-SysMed, Department of Neurology, Haukeland University Hospital, Bergen, Norway, Department of Clinical Medicine, University of Bergen, Bergen, 5021 Bergen, Norway

‡ These authors contributed equally

* These authors contributed equally

Correspondence: marcel.kwiatkowski@uibk.ac.at; r.p.h.bischoff@rug.nl; ines.heiland@uit.no; kathrin.thedieck@uibk.ac.at

Supplemental Figures	P. 2 – 11
Supplemental Tables	P. 12 – 14
Supplemental Experimental Section	P. 15 – 17
Supplemental Tutorial	P. 18 – 26

Supplemental Figures & Table legends

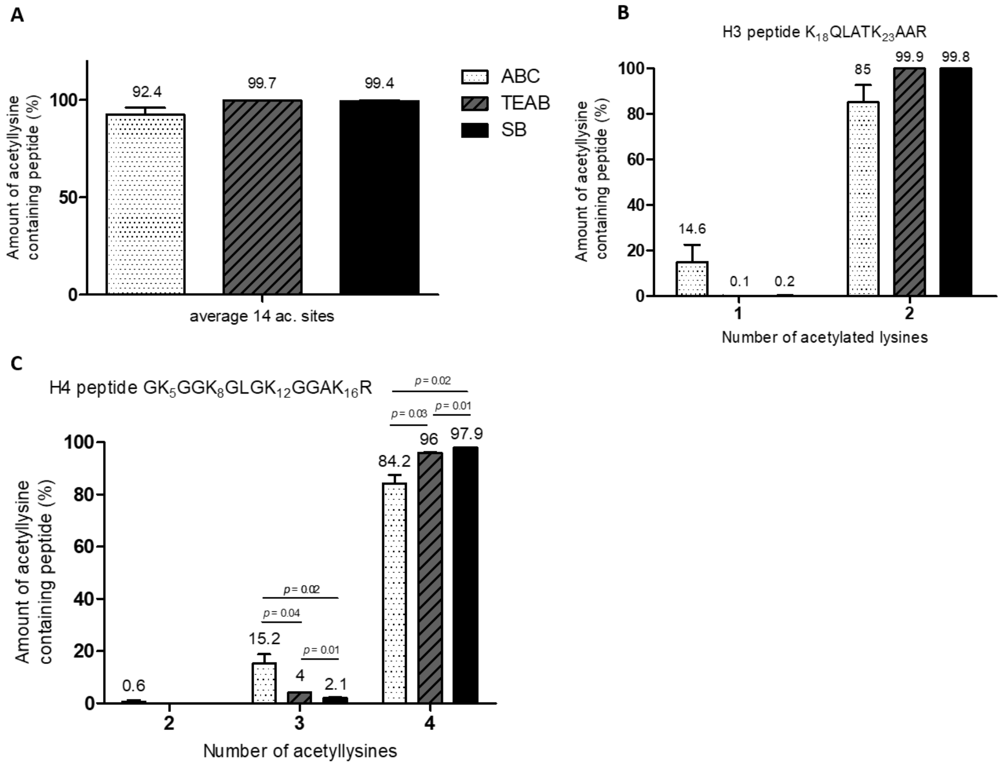

Figure S1: Comparison of the derivatization efficiency of lysine residues with acetic anhydride at the level of intact histone proteins. A: Bar chart (median with SD) showing the total peptide area [%] of 14 derivatized lysine residues of histones H2A, H3 and H4. B, C: Bar charts (median with SD) showing the total peptide area [%] of the different H3(18-26; two acetylation sites) (B) or H4(4-17; four acetylation sites) species (C). ABC: ammonium bicarbonate (c= 100 mM, pH 8.5), TEAB: triethylammonium bicarbonate (c= 100 mM, pH 8.5), SB: sodium borate (c= 100 mM, pH 8.5). n= 3 independent experiments. Statistical analysis: two-tailed unpaired t-test.

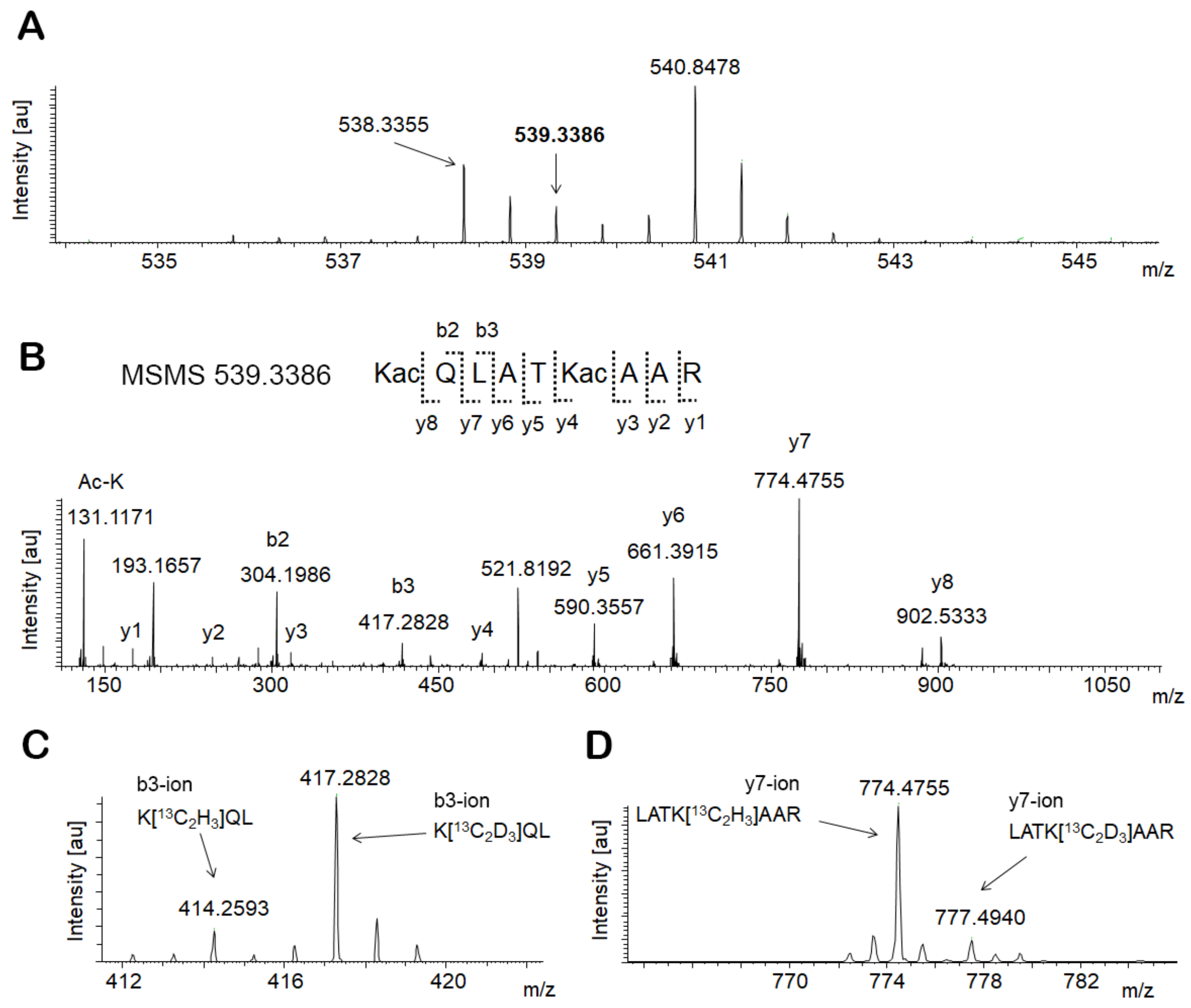

Figure S2: Identification and quantification of site-specific acetylation abundance levels of H3[18-26] positional isomers. The H3[18-26] positional isomers K($^{13}C_2H_3$)QLATK($^{13}C_2D_3$)AAR and K($^{13}C_2D_3$)QLATK($^{13}C_2H_3$)AAR are isobaric and not distinguishable at the MS1 level (A, m/z 539.3386), while they can be distinguished at the MS2 level (B) based on the m/z values of the acetyllysine containing fragment ions such as the b3 (C) and y7 fragment ion (D). The stoichiometry of the two endogenously single-acetylated KQLATKAAR species can be determined based on the relative abundance of the fragment ions. Ac-K: acetyllysine immonium ion ($^{13}C_2D_3$-acetyllysine: m/z 131.1171), $^{13}C_2H_3$: [U-13C]-Glucose derived acetyllysine, $^{13}C_2D_3$: $^{13}C_4D_6$-Acetic anhydride derived acetyllysine.

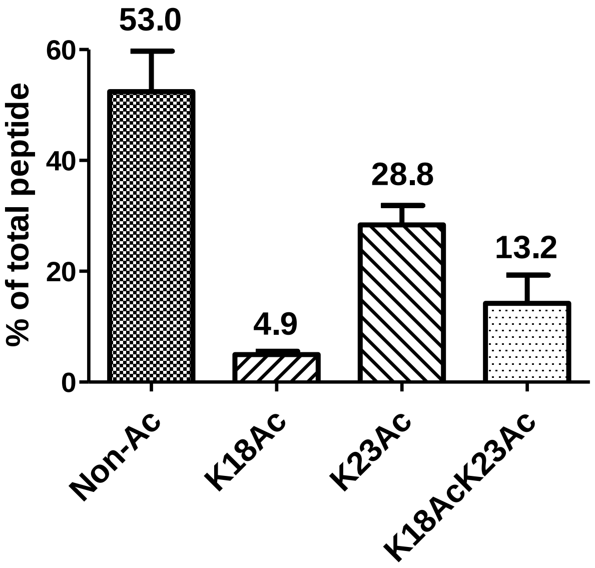

Figure S3: Site-specific abundance of the single- and double-acetylated H3(18-26) peptide species. Bar charts (mean with standard deviation) showing the abundance levels of the non-acetylated (Non-Ac), single-acetylated (K18ac, K23ac) and double-acetylated (K18acK23ac) H3(18-26) species averaged over a time course of 24 hours and the three independent experiments.

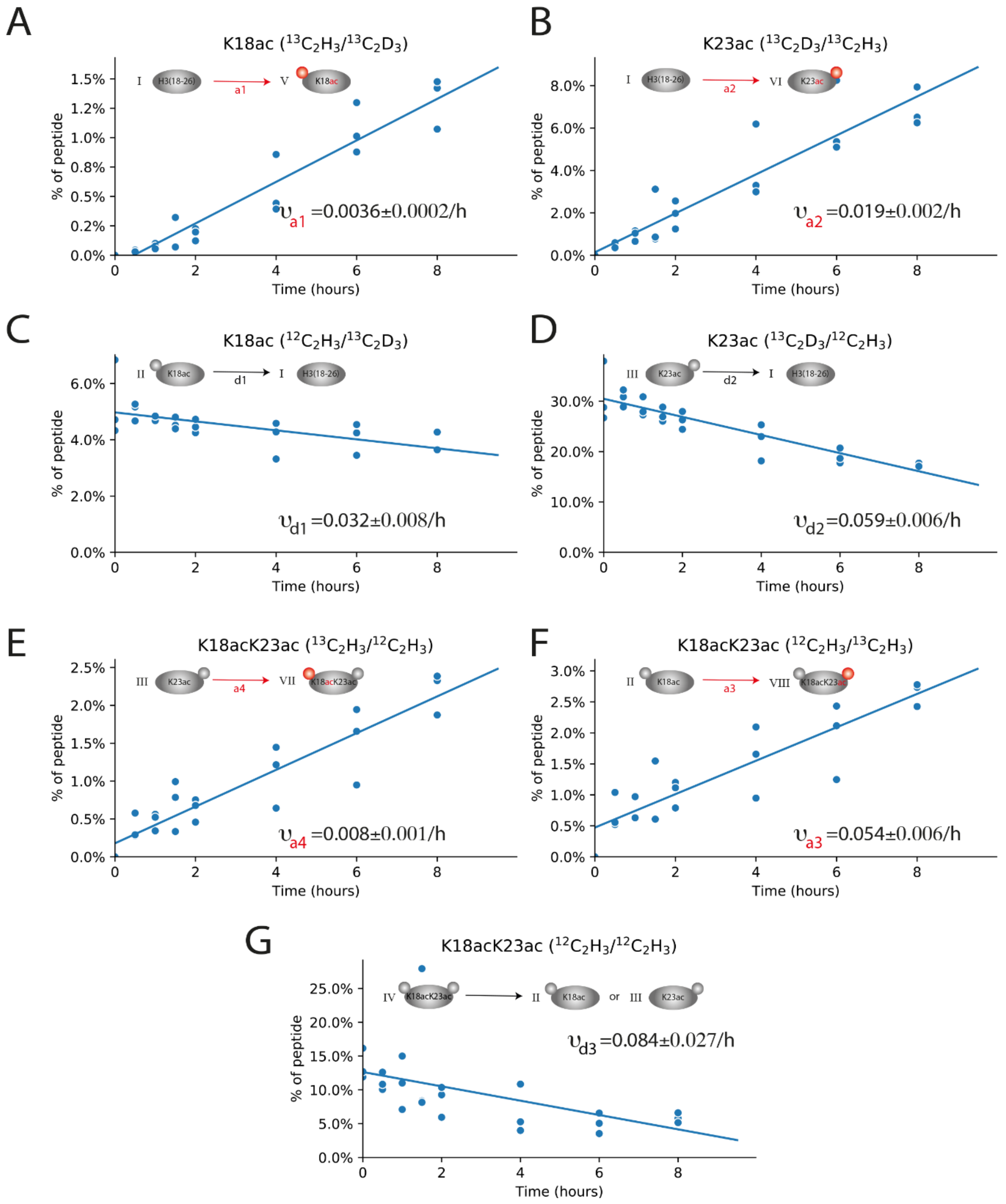

Figure S4: Initial flux analysis to determine reaction rates of acetylation and deacetylation. A-G: Linear fit of the measurements from samples taken within 8 hours after addition of [U-13C]-Glucose used to calculate the acetylation rates of the single-acetylated H3(18-26) species with an acetyllysine at K18ac (A) and K23ac (B), the deacetylation rates at K18ac (C) and K23ac (D); the acetylation rates of the double-acetylated H3(18-26) species with an acetyllysine at K23ac (E) and K18ac (F), and the deacetylation rate of the double-acetylated H3(18-26) species (G).

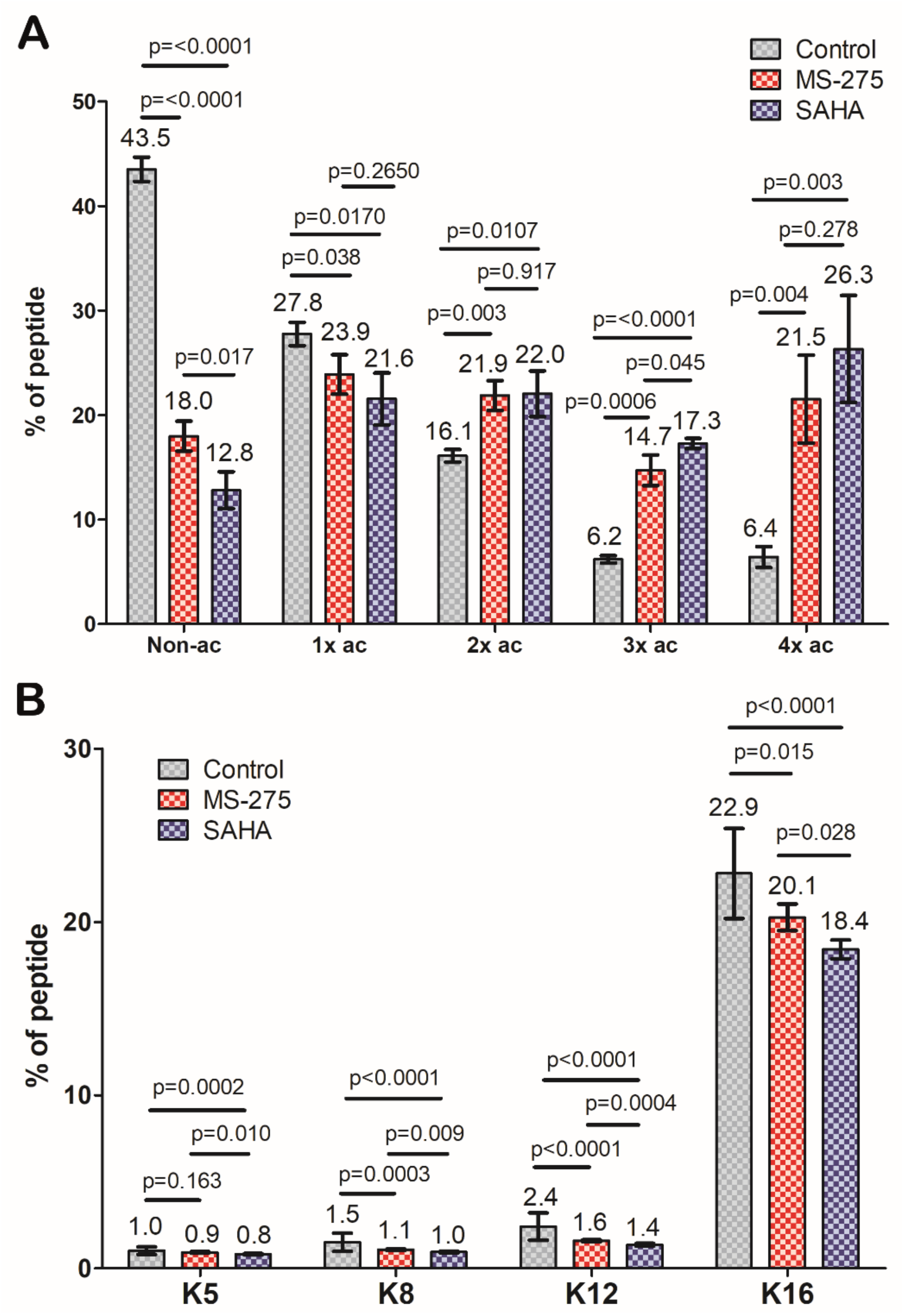

Figure S5: Effect of the histone deacetylase inhibitors MS-275 and SAHA on H4(4-17) acetylation level. A: Bar chart (mean with standard deviation) showing abundance levels of the non-acetylated (Non-ac), single-acetylated (1x ac) and double-acetylated (2x ac), three times acetylated (3x ac) and four times acetylated (4x ac) H4(4-17) species after 16 hours of incubation with MS-275, SAHA or the carrier (control). B: Bar chart (mean with standard deviation) showing site-specific abundance values of each acetylated H4(4-17) species possessing an endogenous acetyllysine at K5, K8, K12, or K16 position after 16 hours of incubation with MS-275, SAHA, or the carrier (control). n = 3 independent experiments. Statistical analyses were performed using the two-tailed unpaired t-test.

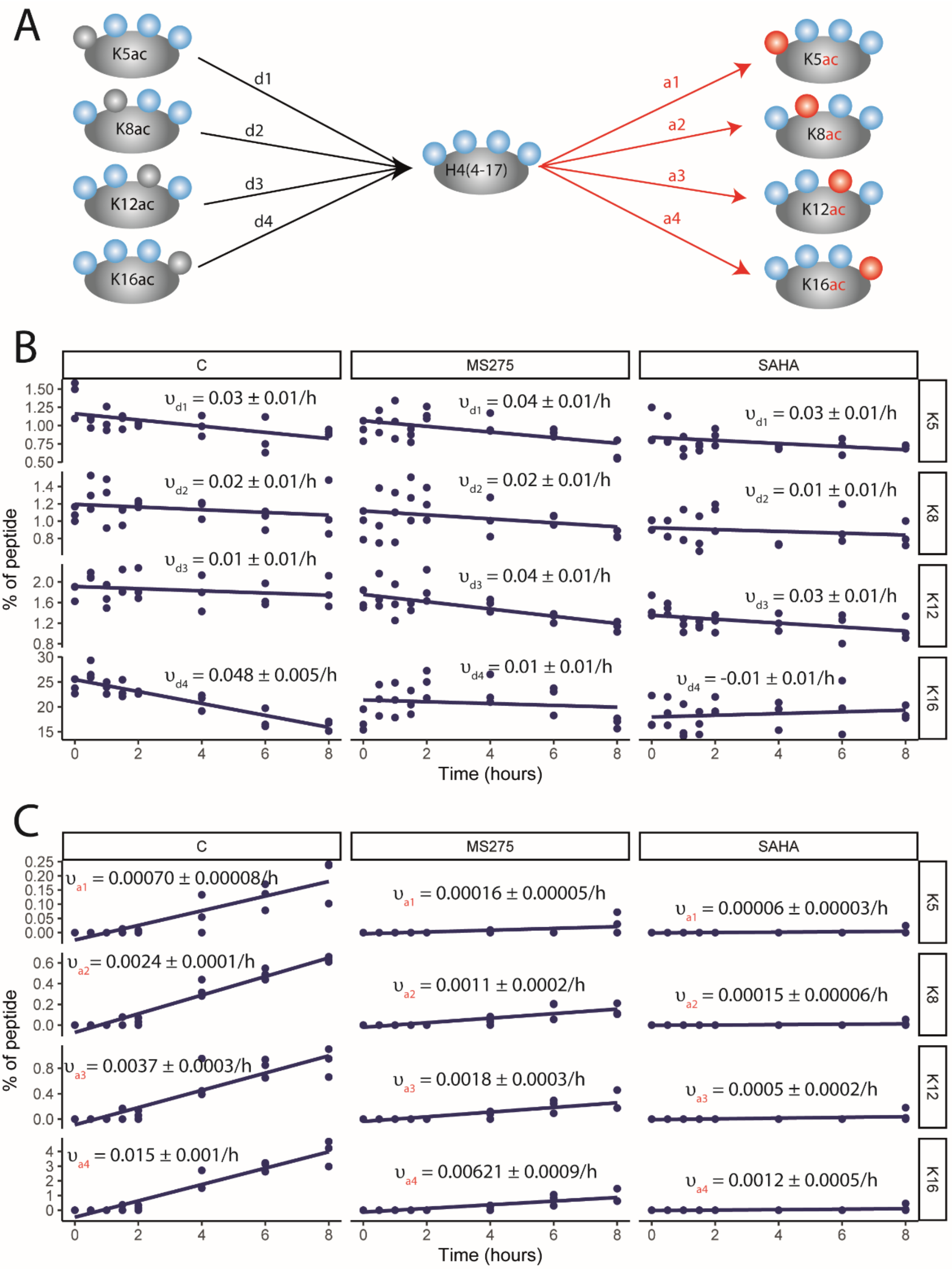

Figure S6: Initial flux analysis to determine reaction rates of acetylation and deacetylation for the single-acetylated H4(4-17) species. A: Schematic representation of possible non-acetylated and single-acetylated H4[4-17] species that can be generated in vivo and reactions that can occur during the metabolic labeling approach with [U-13C]-Glc. Red arrows represent acetylation reactions (marked as ‘’a’’) and black arrows represent deacetylation reactions (marked as ‘’d’’). Acetyl groups derived from [U-12C]-Glc are indicated by gray, [U-13C]-Glc derived acetyl groups are indicated by red, and 13C6,D6-AA derived acetyl groups are indicated by blue circles. B, C: Linear fit of the measurements from samples taken within 8 hours after addition of [U-13C]-Glucose used to calculate the reaction rates of deacetylation (B) and the reaction rates of acetylation (C) of single-acetylated H4(4-17) species with an acetyllysine at K5, K8, K12 or K16 in control samples and after treatment with MS-275 or SAHA.

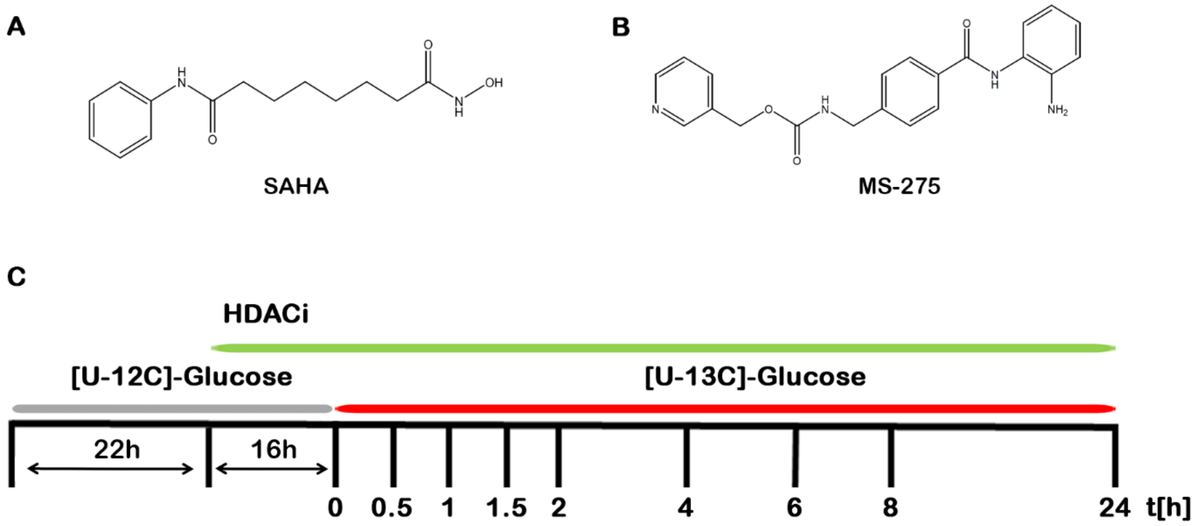

Figure S7: **Chemical structure of the HDAC inhibitors ((SAHA (A), MS-275 (B)) and the workflow for the metabolic labeling (C).** (A) Chemical structure of SAHA (suberanilohydroxamic acid, also known as Vorinostat). (B) Chemical structure of MS-275 (also known as Entinostat). (C) Workflow for the metabolic labeling of the CoMetChem approach to study histone acetylation dynamics upon HDAC inhibitor (HDACi) treatment. RAW264.7 cells were first cultured in a [U-12C]-Glucose containing medium for 22 hours, followed by a pre-incubation of 16 hours with either a histone deacetylase inhibitor (HDACi, MS-275 (c= 1 µM) or SAHA (c= 0.41 µM)) or a carrier (DMSO, c= 0.01%). After pre-incubation, the culture medium was replaced by a [U-13C]-Gluc containing medium including either HDACi or carrier. The nuclei were isolated from the samples at different time points, followed by histone extraction, chemical derivatization of unmodified lysine residues at the protein level using $^{13}C_6$,D_6-acetic anhydride, tryptic digestion and quantitative LC-MS analysis.

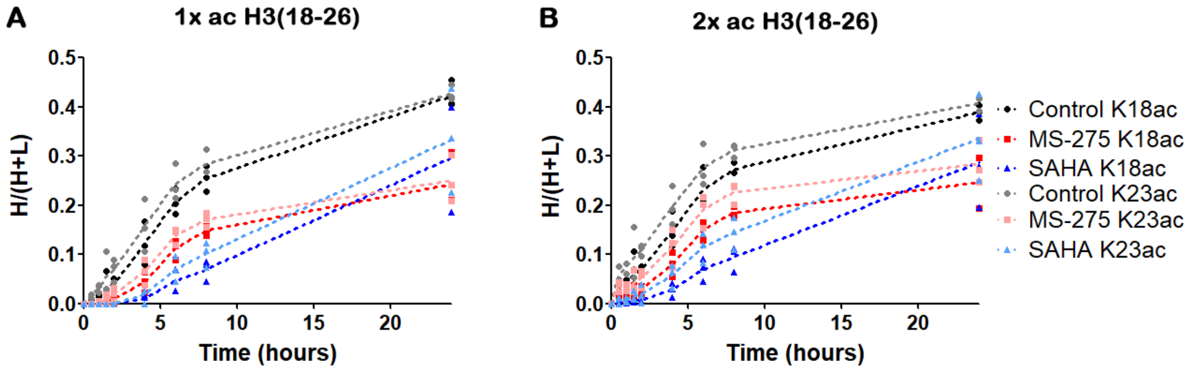

Figure S8: Site-specific label incorporation for K18ac and K23ac of the single acetylated H3(18-26) species (A) and the double-acetylated H3(18-26) species (B). Levels of newly incorporated acetyl groups (% of peptide) were determined by dividing the abundances of the individual acetyllysine-containing peptide species upon [U-13C]-Glucose derived acetylation (H) by the sum of the abundances upon [U-13C]-Glucose (H) and [U-12C]-Glucose (L) derived acetylation.

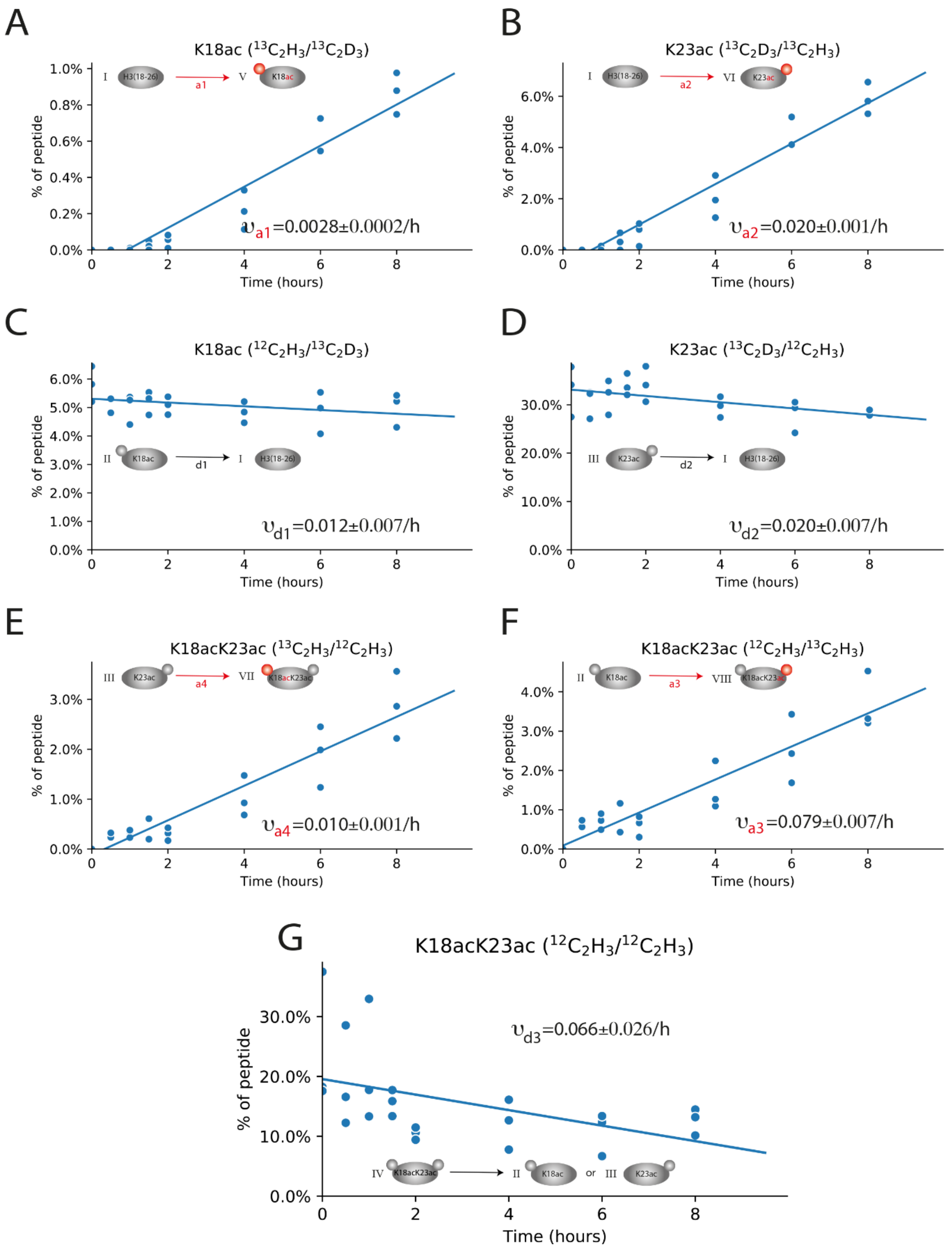

Figure S9: Initial flux analysis to determine reaction rates of acetylation and deacetylation upon MS-275 treatment. Linear fit of the measurements from samples taken within 8 hours after addition of [U-13C]-Glucose used to calculate the acetylation rates of the single-acetylated H3(18-26) species with an acetyllysine at K18ac (A) and K23ac (B), the deacetylation rates at K23ac (C) and K18ac (D); the acetylation rates of the double-acetylated H3(18-26) species with an acetyllysine at K23ac (E) and K18ac (F), and the deacetylation rate of the double-acetylated H3(18-26) species (G).

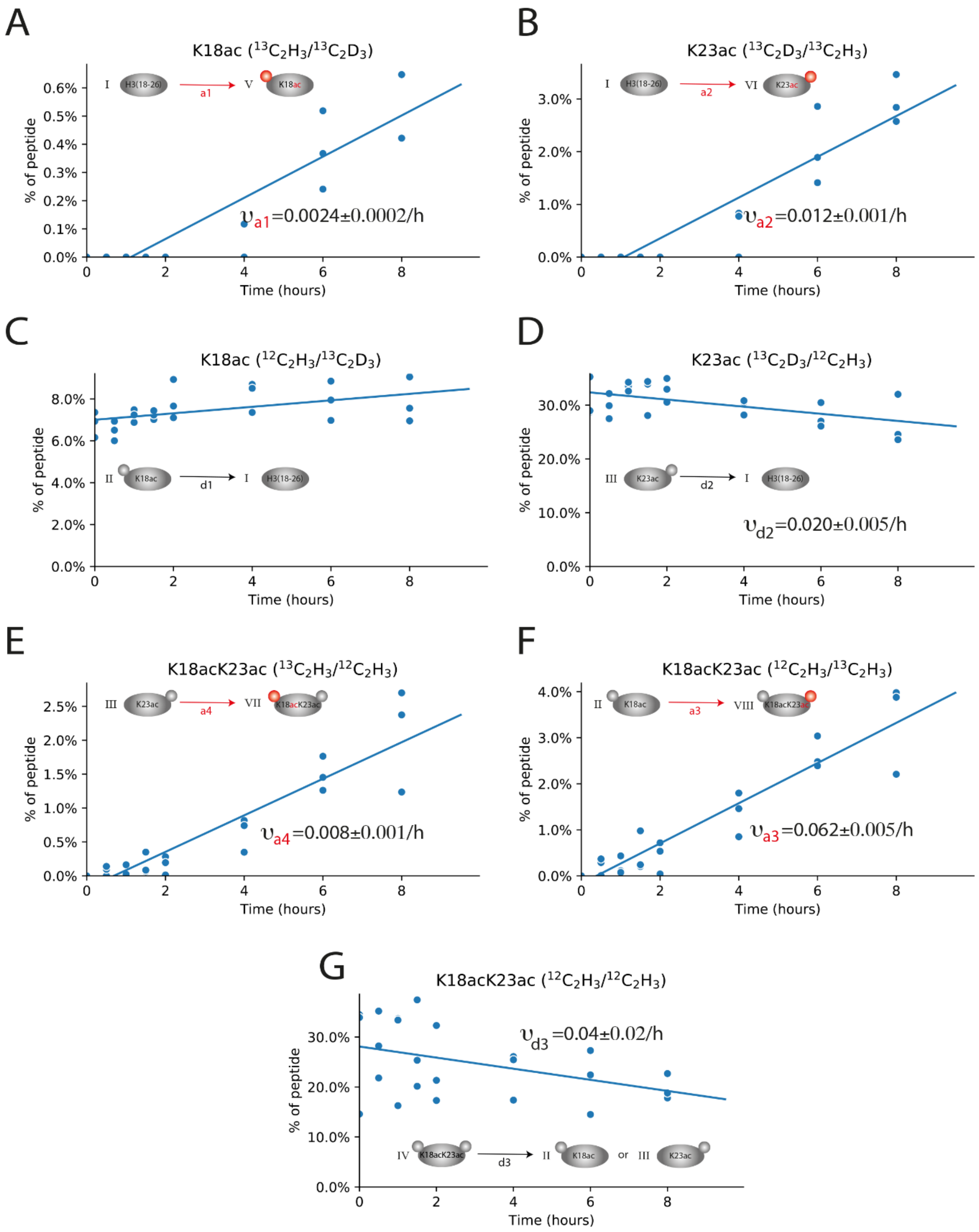

Figure S10: Initial flux analysis to determine acetylation and deacetylation rates upon SAHA treatment. Linear fit of the measurements from samples taken within 8 hours after addition of [U-13C]-Glucose used to calculate the acetylation rates of the single-acetylated H3(18-26) species with an acetyllysine at K18ac (A) and K23ac (B), the deacetylation rates at K23ac (C) and K18ac (D); the acetylation rates of the double-acetylated H3(18-26) species with an acetyllysine at K23ac (E) and K18ac (F), and the deacetylation rate of the double-acetylated H3(18-26) species (G).

Table S1: Calculated half-life times of acetylated H3(18-26) species

The table lists the half-life times that were calculated using exponential fitting of experimental data using the formula $\frac{H}{H+L} = p \cdot (-e^{-kt} + 1)$ as described in the Materials and Methods section. The naming of acetylation sites and isotopologues has been done according to Figure 1-3. The third column refers to the experiment the data are derived from (C for control, MS-275 and SAHA for the inhibitor treatments). In addition to the half-life times, the prefactor p and the exponential prefactor k (the turnover rate) have been listed together with the corresponding standard deviations calculated.

Position	Isotopologue	Exp	half-life	Std	prefactor	std_prefacto	exp_prefacto	std_exp_factor
K23ac	$^{13}C_2D_3/^{12}C_2H_3$ -> $^{13}C_2D_3/^{13}C_2H_3$	C	6h 23min	42min	0.468	0.026	0.108	0.012
K23ac	$^{13}C_2D_3/^{12}C_2H_3$ -> $^{13}C_2D_3/^{13}C_2H_3$	MS275	8h 20min	1h 42min	0.298	0.033	0.083	0.017
K23ac	$^{13}C_2D_3/^{12}C_2H_3$ -> $^{13}C_2D_3/^{13}C_2H_3$	SAHA	21d 10h 9min	287d 14h 2mi	10.000	132.367	0.001	0.018
K18ac	$^{12}C_2H_3/^{13}C_2D_3$ -> $^{13}C_2H_3/^{13}C_2D_3$	C	9h 9min	1h 17min	0.511	0.040	0.076	0.011
K18ac	$^{12}C_2H_3/^{13}C_2D_3$ -> $^{13}C_2H_3/^{13}C_2D_3$	MS275	12h 20min	3h 38min	0.335	0.061	0.056	0.017
K18ac	$^{12}C_2H_3/^{13}C_2D_3$ -> $^{13}C_2H_3/^{13}C_2D_3$	SAHA	24d 23h 44min	470d 5h 32mi	10.000	185.918	0.001	0.022
K18acK23ac	$^{12}C_2H_3/^{12}C_2H_3$ -> $^{12}C_2H_3/^{13}C_2H_3$ & $^{13}C_2H_3/^{12}C_2H$	C	3h 40min	19min	0.578	0.022	0.189	0.016
K18acK23ac	$^{12}C_2H_3/^{12}C_2H_3$ -> $^{12}C_2H_3/^{13}C_2H_3$ & $^{13}C_2H_3/^{12}C_2H$	MS275	4h 40min	35min	0.445	0.027	0.148	0.019
K18acK23ac	$^{12}C_2H_3/^{12}C_2H_3$ -> $^{12}C_2H_3/^{13}C_2H_3$ & $^{13}C_2H_3/^{12}C_2H$	SAHA	1d 0h 16min	11h 8min	0.956	0.332	0.029	0.013

Table S2: Calculated reaction rates of acetylation and deacetylation of the H3(18-26) species

The table lists the reaction rates and the corresponding standard error and R^2 values calculated using the initial flux fitting method described in the Materials and Methods section. The naming of acetylation sites and isotopologues has been done according to Figure 1-3. The reaction names are as indicated in Figure 3 and Supplementary Figures S6 and S7. The fourth column refers to the experiment the data are derived from (C for control, MS-275 and SAHA for the inhibitor treatments).

Position	Chemical label	Reaction	Exp	rate [h^{-1}]	std_err_rate	r_squared
K18acK23ac	$^{12}C_2H_3/^{12}C_2H_3$	d3	C	-0.0839	0.0267	0.3112
K18acK23ac	$^{12}C_2H_3/^{13}C_2H_3$	a3	C	0.0542	0.0062	0.7753
K18acK23ac	$^{13}C_2H_3/^{12}C_2H_3$	a4	C	0.0079	0.0007	0.8594
K23ac	$^{13}C_2D_3/^{12}C_2H_3$	d2	C	-0.0590	0.0064	0.7949
K18ac	$^{12}C_2H_3/^{13}C_2D_3$	d1	C	-0.0321	0.0085	0.3943
K23ac	$^{13}C_2D_3/^{13}C_2H_3$	a2	C	0.0187	0.0015	0.8729
K18ac	$^{13}C_2H_3/^{13}C_2D_3$	a1	C	0.0036	0.0002	0.9221
K18acK23ac	$^{12}C_2H_3/^{12}C_2H_3$	d3	MS275	-0.0663	0.0261	0.2273
K18acK23ac	$^{12}C_2H_3/^{13}C_2H_3$	a3	MS275	0.0793	0.0070	0.8543
K18acK23ac	$^{13}C_2H_3/^{12}C_2H_3$	a4	MS275	0.0104	0.0008	0.8746
K23ac	$^{13}C_2D_3/^{12}C_2H_3$	d2	MS275	-0.0196	0.0073	0.2482
K18ac	$^{12}C_2H_3/^{13}C_2D_3$	d1	MS275	-0.0124	0.0071	0.1205
K23ac	$^{13}C_2D_3/^{13}C_2H_3$	a2	MS275	0.0196	0.0010	0.9423
K18ac	$^{13}C_2H_3/^{13}C_2D_3$	a1	MS275	0.0028	0.0002	0.9160
K18acK23ac	$^{12}C_2H_3/^{12}C_2H_3$	d3	SAHA	-0.0395	0.0182	0.1762
K18acK23ac	$^{12}C_2H_3/^{13}C_2H_3$	a3	SAHA	0.0623	0.0047	0.8885
K18acK23ac	$^{13}C_2H_3/^{12}C_2H_3$	a4	SAHA	0.0083	0.0007	0.8574
K23ac	$^{13}C_2D_3/^{12}C_2H_3$	d2	SAHA	-0.0205	0.0066	0.3031
K18ac	$^{12}C_2H_3/^{13}C_2D_3$	d1	SAHA	0.0221	0.0080	0.2565
K23ac	$^{13}C_2D_3/^{13}C_2H_3$	a2	SAHA	0.0125	0.0011	0.8530
K18ac	$^{13}C_2H_3/^{13}C_2D_3$	a1	SAHA	0.0024	0.0002	0.8283

Table S3 Calculated reaction rates of acetylation and deacetylation of the single-acetylated H4(4-17) species.

The table lists the reaction rates and the corresponding standard error and R^2 values calculated using the initial flux fitting method described in the Materials and Methods section. The naming of acetylation sites and reactions are as indicated in Figure S6 A. The fourth column refers to the experiment the data are derived from (C for control, MS-275 and SAHA for the inhibitor treatments).

Acetylation	Chem label	Reaction	Exp	rate [h^{-1}]	std_err_rate	r_squared
K5ac	$^{12}C_2H_3/^{13}C_2D_3/^{13}C_2D_3/^{13}C_2D_3$	d1	C	0.0312	0.0102	0.3073
K8ac	$^{13}C_2D_3/^{12}C_2H_3/^{13}C_2D_3/^{13}C_2D_3$	d2	C	0.0158	0.0115	0.0832
K12ac	$^{13}C_2D_3/^{13}C_2D_3/^{12}C_2H_3/^{13}C_2D_3$	d3	C	0.0136	0.0098	0.0834
K16ac	$^{13}C_2D_3/^{13}C_2D_3/^{13}C_2D_3/^{12}C_2H_3$	d4	C	0.0480	0.0052	0.8030
K5ac	$^{13}C_2H_3/^{13}C_2D_3/^{13}C_2D_3/^{13}C_2D_3$	a1	C	0.0007	0.0001	0.7807
K8ac	$^{13}C_2D_3/^{13}C_2H_3/^{13}C_2D_3/^{13}C_2D_3$	a2	C	0.0024	0.0001	0.9468
K12ac	$^{13}C_2D_3/^{13}C_2D_3/^{13}C_2H_3/^{13}C_2D_3$	a3	C	0.0037	0.0003	0.8460
K16ac	$^{13}C_2D_3/^{13}C_2D_3/^{13}C_2D_3/^{13}C_2H_3$	a4	C	0.0151	0.0010	0.9193
K5ac	$^{12}C_2H_3/^{13}C_2D_3/^{13}C_2D_3/^{13}C_2D_3$	d1	MS275	0.0362	0.0122	0.2861
K8ac	$^{13}C_2D_3/^{12}C_2H_3/^{13}C_2D_3/^{13}C_2D_3$	d2	MS275	0.0206	0.0146	0.0830
K12ac	$^{13}C_2D_3/^{13}C_2D_3/^{12}C_2H_3/^{13}C_2D_3$	d3	MS275	0.0401	0.0101	0.4192
K16ac	$^{13}C_2D_3/^{13}C_2D_3/^{13}C_2D_3/^{12}C_2H_3$	d4	MS275	0.0085	0.0124	0.0209
K5ac	$^{13}C_2H_3/^{13}C_2D_3/^{13}C_2D_3/^{13}C_2D_3$	a1	MS275	0.0002	0.0001	0.3030
K8ac	$^{13}C_2D_3/^{13}C_2H_3/^{13}C_2D_3/^{13}C_2D_3$	a2	MS275	0.0011	0.0002	0.6907
K12ac	$^{13}C_2D_3/^{13}C_2D_3/^{13}C_2H_3/^{13}C_2D_3$	a3	MS275	0.0018	0.0003	0.6966
K16ac	$^{13}C_2D_3/^{13}C_2D_3/^{13}C_2D_3/^{13}C_2H_3$	a4	MS275	0.0062	0.0009	0.7097
K5ac	$^{12}C_2H_3/^{13}C_2D_3/^{13}C_2D_3/^{13}C_2D_3$	d1	SAHA	0.0255	0.0131	0.1469
K8ac	$^{13}C_2D_3/^{12}C_2H_3/^{13}C_2D_3/^{13}C_2D_3$	d2	SAHA	0.0113	0.0138	0.0293
K12ac	$^{13}C_2D_3/^{13}C_2D_3/^{12}C_2H_3/^{13}C_2D_3$	d3	SAHA	0.0280	0.0109	0.2318
K16ac	$^{13}C_2D_3/^{13}C_2D_3/^{13}C_2D_3/^{12}C_2H_3$	d4	SAHA	-0.0096	-0.0125	0.0264
K5ac	$^{13}C_2H_3/^{13}C_2D_3/^{13}C_2D_3/^{13}C_2D_3$	a1	SAHA	0.0001	0.0000	0.1592
K8ac	$^{13}C_2D_3/^{13}C_2H_3/^{13}C_2D_3/^{13}C_2D_3$	a2	SAHA	0.0001	0.0001	0.2108
K12ac	$^{13}C_2D_3/^{13}C_2D_3/^{13}C_2H_3/^{13}C_2D_3$	a3	SAHA	0.0005	0.0002	0.1957
K16ac	$^{13}C_2D_3/^{13}C_2D_3/^{13}C_2D_3/^{13}C_2H_3$	a4	SAHA	0.0012	0.0005	0.2011

Supplemental Experimental Section

Chemicals

Water, acetonitrile (ACN), formic acid (FA), triethylammonium bicarbonate, dimethylsulfoxide (all HPLC-grade), Micro BCA™ Protein Assay Kit, Gibco® Qualified FBS were obtained from Thermo Fisher Scientific (Dreieich, Germany). MS-275 and SAHA were obtained from Selleckchem (Munich, Germany). [U-13C]-Glucose and $^{13}C_4$,D_6-acetic anhydride were purchased from (Euroisotope, Saarbrücken, Germany). Sodium borate and ammonium bicarbonate, dithiothreitol, iodoacetamide, sodium butyrate, nicotinamide, phenylmethylsulfonyl fluoride were obtained from Sigma-Aldrich (Munich, Germany). Dulbecco's Modified Eagle Medium was purchased from PAN Biotech (Aidenbach, Germany). All other chemicals were purchased from Sigma-Aldrich (Munich, Germany) unless otherwise noted.

Cell culture and metabolic labeling with [U-13C]-glucose

The murine macrophage cell line RAW264.7 (American Type Culture Collection, Wesel, Germany) was cultured in TPP® tissue culture 6 well plates (M&B Stricker, Bernried, Germany) at 37°C and 5% CO2 atmosphere in DMEM with 4.5 g/L glucose. Media was supplemented with 2 mM extra L-Glutamine and 10% (v/v) heat-inactivated FBS. To investigate the efficiency of chemical acetylation, cells were cultured in medium containing [U-12C]-glucose (([U-12C]-Glc) for 48 hours. For metabolic labeling, RAW264.7 cells were first cultured in [U-12C]-Glc) containing medium for 22 hours, followed by a pre-incubation of 16 hours with either a histone deacetylase inhibitor (HDACi, MS-275 (c= 1 µM, dissolved in 0.01% DMSO) or suberanilohydroxamic acid (SAHA, c= 0.41 µM, dissolved in 0.01% DMSO)) or a carrier (DMSO, c= 0.01%) in [U-12C]-Glc containing medium. After pre-incubation, the culture medium was replaced by a [U-13C]-Glucose ([U-13C]-Glc) containing medium including either MS-275, SAHA or carrier. The cells were grown for 24 hours.

Histone extraction

For histone extraction, the cell nuclei were isolated first. The cells were collected and washed three times with PBS. For lysis, 500 µL ice-cold nuclear isolation buffer (15mM Tris-HCl, 15 mM NaCl, 60 mM KCl, 5 mM MgCl2, 1 mM CaCl2, 250 mM sucrose, 1mM dithiothreitol (DTT), 1mM phenylmethylsulfonyl fluoride (PMSF), 10mM nicotinamide, and 10mM sodium butyrate, 0.01% sodium deoxycholate(SDC)) was used. The homogenized cells were incubated for 10 min on ice and washed three times with the nuclear isolation buffer without SDC. Histones were extracted from the nuclei using 0.2 M hydrogen chloride (HCl) at a cell density of 8 × 103 cells/µL and samples were incubated on a rotator at 4°C for 30 min. Afterwards, the samples were centrifuged (16 000× g, 10 min, 4°C). The supernatants were transferred to a new reaction tube and trichloroacetic acid was slowly added to a final concentration of 25% (w/v). The reaction tubes were inverted five times and incubated for 35 min on ice. The histones were pelleted by centrifugation (16 000 × g, 10 min, 4°C) and washed three times with ice-cold acetone. The samples were air-dried in a fume hood for 30 min at RT. The histone pellets were dissolved in 50 µL H2O (HPLC grade). Protein concentration was determined using the microplate BCA protein assay kit following the manufacturer's instructions. A BSA standard (2 mg/mL) was used to calibrate the assay across the concentration range of 2-40 µg/mL. The absorbance was measured at 580 nm using a plate reader.

Chemical acetylation using 13C4,D6-acetic anhydride and tryptic digestion to investigate chemical acetylation efficiency

To study the chemical acetylation efficiency, 6 µg of the histone extract were diluted in HPLC-H_2O to a final volume of 50 µL. For reduction, the samples were incubated with 10 mM DTT (dissolved in 100 mM sodium borate (SB, pH 8.5), 100 mM ammonium bicarbonate (ABC, pH 8.5) or triethylammonium bicarbonate (TEAB, pH 8.5) for 10 min at 57°C on a shaker (600 rpm). Afterwards, the samples were alkylated with 20 mM iodoacetamide (IAA, dissolved in 100 mM SB, 100 mM ABC or 100 mM TEAB, pH 8.5) and incubated for 30 min at room temperature in the dark. Subsequently, free IAA was quenched by adding 10 mM of DTT (dissolved in 100 mM SB, 100 mM ABC or 100 mM SB, pH 8.5). Subsequently, 50 µL of 100 mM SB (pH 8.5), 100 mM ABC (pH 8.5) or 100 mM TEAB (pH 8.5) were

added. Then 1 µL of 4.1 mM 13C4,D6-acetic anhydride (13C4,D6-AA, dissolved in water-free DMSO) was added, followed by incubation on a thermomixer (1000 rpm) at 25°C for 10 minutes. The chemical acetylation was repeated twice. O-acetylation was reverted by adding hydroxylamine to final concentration of 7.6 mM, followed by an incubation on a thermomixer (450 rpm) at 25°C for 120 minutes. For tryptic digestion, the samples were incubated with 1 µL of a trypsin solution (c = 0.2 µg/µL sequencing grade modified trypsin, dissolved in trypsin resuspension buffer, Promega, Walldorf, Germany) for 16 hours at 37°C. Afterwards, the samples were acidified by adding formic acid (FA) to final concentration of 0.1% FA.

LC-MS/MS analysis of chemically acetylated histone species and bioinformatics data processing

LC-MS/MS measurements were performed by injecting the samples on a nano-ultra pressure liquid chromatography system (Dionex UltiMate 3000 RSLCnano, Thermo Scientific, Bremen, Germany) coupled via electrospray-ionization (ESI) to a quadrupole orbitrap mass spectrometer (Orbitrap QExcactive Plus, Thermo Scientific, Bremen, Germany). The samples were loaded (20 µL/min) on a trapping column (Acclaim PepMap µ-precolumn, C18, 300 µm x 5 mm, 5 µm, 100 Å, Thermo Scientific, Bremen, Germany; nanoACQUITY UPLC Symmetry C18 trap column, 180 µm × 20 mm, 5 µm, 100 Å; buffer A: 0.1% FA in HPLC-H2O; buffer B: 0.1% FA in ACN) with 2% buffer B. After sample loading the trapping column was washed for 5 minutes with 2% buffer B (5 µL/min) and the peptides were eluted (300 nL/min) onto the separation column (Acclaim PepMap 100, C18, 75 µm × 500 mm, 2 µm, 100 Å, Thermo Scientific, Bremen, Germany) and separated with a gradient from 2−30% B in 30 min. The spray was generated from a steel emitter (I.D. 30 µm, Proxeon, Thermo Scientific, Bremen, Germany) at a capillary voltage of 1850 V. Mass spectrometric analyses were performed in positive ion mode. LC-MS/MS analysis was performed at the MS level over an m/z range from 400-1500, with a resolution of 70000 FWHM at m/z 200 (transient length= 256 ms, injection time= 30 ms, AGC target= 1e6). MS/MS measurements were carried out in DDA mode (loop count: 10), with an HCD collision energy of 25%, a resolution of 17,500 FWHM at m/z 200 (transient length= 64 ms, injection time = 120 ms, minimum AGC target= 2e3), an isolation width of 1.6 m/z and a dynamic exclusion of 30 s.

LC-MS/MS raw data were processed and quantified with MaxQuant (version 1.5.4.1). Peptide and protein identification was carried out with Andromeda against a murine SwissProt database (16,844 entries) and a contaminant database (115 entries). The searches were performed using the following parameters: precursor mass tolerance was set to 6 ppm and fragment mass tolerance was set to 20 ppm. For peptide identification, five missed cleavages were allowed. Carbamidomethylation of cysteine residues was considered as a fixed modification. Oxidation of methionine residues and an acetylation of lysine residues (light: H(2) C(2) O, heavy: D(3) C(2) O) were allowed as variable modifications. Peptides and proteins were identified with an FDR of 1%. For analysis of chemical acetylation efficiencies, peptide intensities of the “modification_specific_peptide” output file were used. Statistical analysis was performed with GraphPad Prism (version 5) using two-tailed unpaired t-test.

LC-MS/MS analysis of CoMetChem labeled histone species

For LC-MS/MS analysis, 100 ng of the tryptic peptide digests were injected on a nano-ultra pressure liquid chromatography system (Dionex UltiMate 3000 RSLCnano pro flow, Thermo Scientific, Bremen, Germany) coupled via an electrospray ionization (ESI) source to a tribrid orbitrap mass spectrometer (Orbitrap Fusion Lumos, Thermo Scientific, San Jose, CA, USA). The samples were loaded (15 µL/min) on a trapping column (nanoE MZ Sym C18, 5µm, 180umx20mm, Waters, Germany, buffer A: 0.1% FA in HPLC-H2O; buffer B: 80% ACN, 0.1% FA in HPLC-H2O) with 5% buffer B. After sample loading, the trapping column was washed for 2 min with 5% buffer B (15 µL/min) and the peptides were eluted (250 nL/min) onto the separation column (nanoE MZ PST CSH, 130 A, C18 1.7µ, 75µmx250mm, Waters, Germany) and separated with a gradient of 5−37.5% B in 25 min, followed by 37.5-62.5% in 5 min. The spray was generated from a steel emitter (Fisher Scientific, Dreieich, Germany) at a capillary voltage of 1900 V. MS/MS measurements were carried out in data dependent acquisition mode (DDA) using an HCD collision energy of 30% and two different scan modes. Scan mode 1: MS/MS spectra were acquired in top speed mode. Every second an MS scan was performed over an m/z range from 350-1600, with a resolution of 60,000 FWHM at m/z 200 (maximum

injection time= 50 ms, AGC target= 4e5) using fluoranthene for internal calibration. MS/MS spectra were recorded in the orbitrap with a resolution of 7500 FWHM at m/z 200 (maximum injection time= 50 ms, AGC target= 5e5, quadrupole isolation width: 0.8). Scan mode 2: For isobaric H3(18-26) isotopologues, an inclusion list with a defined m/z (+/- 5 ppm), charge state and retention time window was used (m/z 536.8228, z=2; m/z 538.3322, z=2; m/z 539.3356, z=2, RT: 22.07-23.07 min). MS/MS spectra were recorded in the orbitrap with a resolution of 7,500 FWHM at m/z 200 (Scan mode 1: maximum injection time= 50 ms, AGC target= 100%, intensity threshold: 2.5e4 quadrupole isolation width: 0.4 Da, Scan mode 2: maximum injection time= 100 ms, AGC target= 100%, intensity threshold: 5e5, quadrupole isolation width: 0.4 Da).

MS2 fragment ion quantification using PASTAQ

Raw-files were converted to mzML-files using msconvert (http://proteowizard.sourceforge.net/tools.shtml) and MS2 spectra were extracted using PASTAQ's [1] Python bindings. The resulting MS/MS spectra were filtered to only contain those with precursor m/z and retention time within the given min/max retention time window for each inclusion list entry. Each MS/MS spectrum was searched for the theoretical fragments of interest by extracting a slice of data centered around the theoretical m/z. Finally, the fragments were quantified by performing a weighted Gaussian curve fitting using non-linear least squares and the area under the curve was calculated using the trapezoidal rule and exported together with the raw total intensity, fitted height, fitted m/z, and fitted width of the peak. For site-specific quantification of isobaric H3(18-26) isotopologue species, the relative MS2 abundance of the isotopologue species is multiplied with the corresponding MS1 abundance. For site-specific quantification of the single-acetylated H4(4-17) species, the intensities for the y5, y7, and y12 ions of the single-acetylated precursors were quantified and the relative abundances were calculated according to the approach of Feller et al. [2] as follows:

$$\mathrm{H4_{K5}} = \frac{\mathrm{Int.}\,(\mathrm{MS1_{1xac\ H4(4-17)}})}{\Sigma\ \mathrm{Int}(\mathrm{MS1_{totalH4(4-17)}})} \cdot \left(1 - \frac{\mathrm{y12_{Light1xacH4(4-17)}}}{\left(\mathrm{y12_{Light1xacH4(4-17)}} + \mathrm{y12_{heavy1xacH4(4-17)}}\right)}\right)$$

$$\mathrm{H4_{K8}} = \frac{\mathrm{Int.}\,(\mathrm{MS1_{1xac\ H4(4-17)}})}{\Sigma\ \mathrm{Int}(\mathrm{MS1_{totalH4(4-17)}})} \cdot \left(\frac{\mathrm{y12_{Light1xacH4(4-17)}}}{\left(\mathrm{y12_{Light1xacH4(4-17)}} + \mathrm{y12_{heavy1xacH4(4-17)}}\right)} - \frac{\mathrm{y7_{Light1xacH4(4-17)}}}{\left(\mathrm{y7_{Light1xacH4(4-17)}} + \mathrm{y7_{heavy1xacH4(4-17)}}\right)}\right)$$

$$\mathrm{H4_{K12}} = \frac{\mathrm{Int.}\,(\mathrm{MS1_{1xac\ H4(4-17)}})}{\Sigma\ \mathrm{Int}(\mathrm{MS1_{totalH4(4-17)}})} \cdot \left(\frac{\mathrm{y_{7_{Light1xacH4(4-17)}}}}{\left(\mathrm{y_{7_{Light1xacH4(4-17)}}} + \mathrm{y_{7_{heavy1xacH4(4-17)}}}\right)} - \frac{\mathrm{y_{5_{Light1xacH4(4-17)}}}}{\left(\mathrm{y_{5_{Light1xacH4(4-17)}}} + \mathrm{y_{5_{heavy1xacH4(4-17)}}}\right)}\right)$$

$$\mathrm{H4_{K16}} = \frac{\mathrm{Int.}\,(\mathrm{MS1_{1xac\ H4(4-17)}})}{\Sigma\ \mathrm{Int}(\mathrm{MS1_{totalH4(4-17)}})} \cdot \frac{\mathrm{y_{5_{Light1xacH4(4-17)}}}}{\left(\mathrm{y_{5_{Light1xacH4(4-17)}}} + \mathrm{y_{5_{heavy1xacH4(4-17)}}}\right)}$$

Supplemental Tutorial – CoMetChem data analysis pipeline

In the following section, a detailed tutorial on bioinformatics workflow (Figure S11) and data processing can be found. The different steps of the pipeline are exemplarily explained by the H3(18-26) peptide KQLATKAAR.

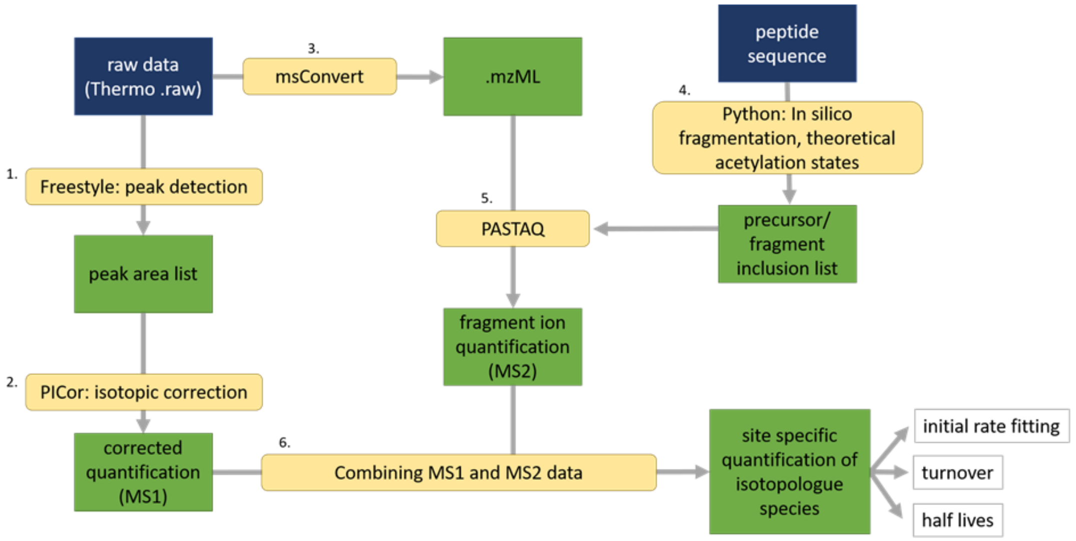

Figure S11: **Flow-chart showing the different steps of the CoMetChem data-analysis pipeline**. Initial input files are indicated in blue boxes, programs/scripts are indicated in yellow boxes, output files are indicated in green boxes.

1. Generation of extracted ion chromatograms (EICs) of the H3(18-26) isotopologue species

Extracted ion chromatograms (EICs) of the isotopologue species have to be generated for the analysis. For this, the LC-MS raw data (*.raw) is analyzed by FreeStyle (Thermo Fisher Scientific). FreeStyle can be downloaded under the Life Sciences Mass Spectrometry Software Download and Licensing Portal of Thermo Fisher Scientific (https://thermo.flexnetoperations.com/control/thmo/login).

1.1 Load LC-MS raw data file (*.raw) in Freestyle 1.6 (Version 1.6.75.20, Thermo Scientific, Bremen, Germany).

1.2 Evaluate the MS1 spectrum of the different acetylated H3(18-26) isotopologue species (Figure S12).

1.3 To generate extracted ion chromatograms (EIC) of the different H3(18-26) isotopologue species (Figure S13) the following parameters are used in the "chromatogram ranges" tab:
Detector Type: MS
Trace Type: Mass Range
Ranges: theoretical m/z of the corresponding isotopologue species
Mass Tolerance: 3 ppm (Note: The mass tolerance should be adjusted according to the measurement accuracy).

1.4 Export the workspace of peak detection including peak heights and peak areas of the EICs as *.csv file (see example Table S4).

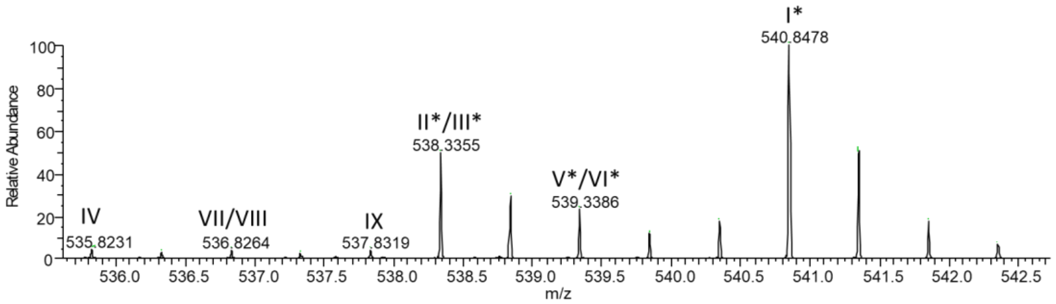

Figure S12: MS1 spectrum of the H3(18-26) isotopologues upon CoMetChem labeling after 4h of incubation with [U-13C]-Glc. Monoisotopic peaks of the unacetylated H3(18-26) (m/z 540.8478), single-acetylated H3(18-26) species (m/z 538.3355 and m/z 539.3386), and double-acetylated H3(18-26) species (535.8231, 536.8264, and 537.8319) are indicated with Roman numerals, consistent with Figure 1C in the main manuscript.

A

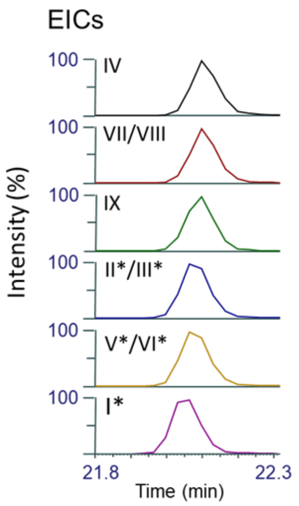

B

H3 (18-26) isotopologue	acetyl group compositions	Theoretical m/z
IV	$^{12}C_2H_3/^{12}C_2H_3$	535.8195
VII/VIII	$^{12}C_2H_3/^{13}C_2H_3$	536.8228
IX	$^{13}C_2H_3/^{13}C_2H_3$	537.8262
II*/III*	$^{12}C_2H_3/^{13}C_2D_3$	538.3322
V*/VI*	$^{13}C_2H_3/^{13}C_2D_3$	539.3356
I*	$^{13}C_2D_3/^{13}C_2D_3$	540.845

Figure S13: Extracted ion chromatograms (EICs) of the H3(18-26) isotopologue species. Monoisotopic peaks of the unacetylated peptide (m/z 540.8478), single-acetylated peptide species (m/z 538.3355 and m/z 539.3386), and double-acetylated peptide species (535.8231, 536.8264, and 537.8319) are indicated with Roman numerals, consistent with main figure 1C. Extracted ion chromatograms (A) and acetyl group compositions and theoretical m/z of the H3(18-26) isotopologue species (B).

Table S4: Intensities of the different isotopologue species of the H3(18-26) peptide. Peak heights obtained from the extracted ion chromatograms of the monoisotopic peaks of the H3(18-26) isotopologue species.

Time (h)	IV	VII/VIII	IX	II*/III*	V*/VI*	I*	Rep	Exp
4	3.14E+07	2.45E+07	1.86E+07	1.76E+08	7.91E+07	3.45E+08	1	C

2. Natural stable isotope correction using PICor

To quantify the different isotopologue species, a correction of measured isotope intensities for naturally occurring stable isotopes is performed using PICor. The python PICor package and source code is available under https://github.com/MolecularBioinformatics/PICor. The detailed approach of PICor is described on bioRxiv [3].

2.1 Read in *.csv file containing the intensities of the EICs of the isotopes of the H3(18-26) isotopologue species MS1 data (see section 1). The columns containing intensities have to be renamed so they contain information about the number of isotopically labeled atoms (e.g. 2C13 3H02 for the species containing one chemical acetylation).

2.2 Generate a *.tsv containing the name, sum formula and charge of your analyte of interest (Note: the sum formula has to contain all atoms including the acetyl group of the acetyllysine residues) (Table S5).

Table S5: Examplary input for the H3(18-26) peptide for PICor correction.

name	formula	charge
K(ac)QLATK(ac)AAR	C46H84N15O14	+2

2.3 Run the python PICor package from the command line using the following command:

“picor input.csv -o outfile.csv -x Rep -x Exp --molecules-file peptides.tsv -r --resolution 60000 --mz-calibration 200”¬

Where Rep and Exp are column names that do not contain intensity values and should be excluded. The input file containing intensity values is generated in section 2.1, the peptides.tsv file was generated in section 2.2.

Figure S14 shows the heights of the different isotopologue species before (upper graph) and after (lower graph) PICor correction.

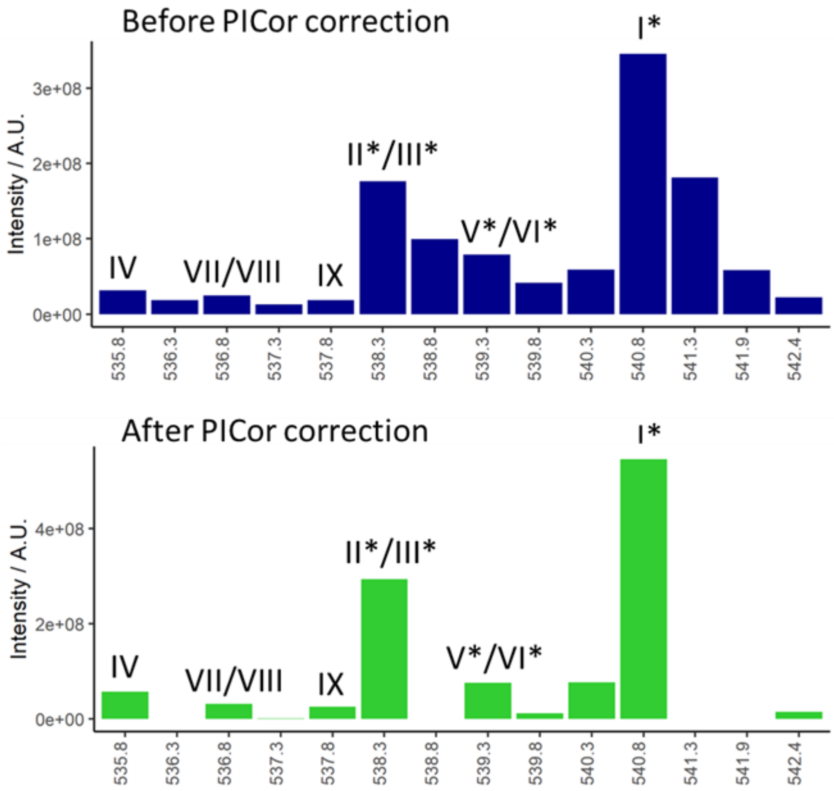

Figure S14: Abundances of the six different distinguishable isotopologue species of the H3(18-26) peptide KQLATKAAR before (upper panel) and after (lower panel) PICor correction. Monoisotopic peaks of the unacetylated peptide (m/z 540.8450), singly acetylated peptide species (m/z 538.3322 and m/z 539.3356), and doubly acetylated peptide species (535.8195, 536.8228, and 537.8262) are indicated with Roman numerals, consistent with main figure 1C. To visualize the isotopic correction, the M+1, M+2 and M+3 peaks of the different monoisotopic peaks are also included.

3. Raw data file conversion using msConvert

The single-acetylated H3(18-26) isotopologue species II*/II* and V*/VI*, as well as the double-acetylated species VII*/VIII* are positional isomers and isobaric at MS1 level but are distinguishable based on their fragment ion spectra. To detect the isotopologue-specific fragment ions by PASTAQ

(see section 5), the LC-MS raw data files (*.raw) have to be converted into *.mzML. For data conversion the program msConvert is used which can be downloaded via http://proteowizard.sourceforge.net/download.html.

3.1 Load *.raw input files
3.2 Disable zlib compression
3.3 Set an output directory
3.4 Start the conversion process

4 Calculation of isotopologue species-specific fragment ions

For the quantification of isotopologue-specific fragment ions with PASTAQ (see Section 5), the m/z values of the y- and b-fragment ions of the individual isotopologue species have to be calculated. For this we use an in-house generated Python script, which can be downloaded at https://github.com/functional-proteomics-and-metabolomics/CoMetChem as Fragmentation.py. For detailed information about the script see the corresponding readme file.

4.1 In the Python file set the possible modifications (ac12: 12C2H3O -H; ac13: 13C2H3O -H; ac*: 13C2D3O -H) and the peptide sequence (single letter amino acid code) as DICT_MODIFICATIONS and SEQUENCE, respectively.
4.2 Run the script.
4.3 Two *.csv-files are generated. SEQUENCE_Fragmentaion.csv includes all possible fragments, whereas SEQUENCE_precursorSpecificIon.csv includes all ions capable for identification and quantification of a specific species.
4.4 The python script generates a list with all theoretically possible fragment ions with the following outputs: theoretical m/z (z= 2) of the intact isotopologue species(precursor m/z); type of fragment ion (b-ions, y-ions); theoretical m/z of the fragment ions; amino acid sequence (one letter code) of the fragment ion including the type of acetyl group of the acetyllysine residues.
4.5 Export isotopologue species-specific fragment ion information as a .csv file.

5 MS2 fragment ion quantification using PASTAQ

To determine site-specific acetylation levels for MS1 isobaric isotopologue species, the relative abundances of site-specific acetyllysine containing fragment ions were quantified using Pipelines and Systems for Threshold Avoiding Quantification of LC-MS/MS data (PASTAQ)[1]. PASTAQ can be downloaded under https://pastaq.horvatovichlab.com/.

5.1 For MS2 fragment quantification analysis with PASTAQ, a sample table must be generated (*.csv). The sample table (see Table S6) has to include the following information:
sample name: Sample name identical to the LC-MS raw data input file (*.mzML).
precursor_min_rt: Starting time point [min] of the retention window of the precursor ion.
precursor_min_rt: End time point [min] of the retention window of the precursor ion.
precursor_mz: The theoretical m/z of the precursor of the isotopologue species (calculated in section 4).
precursor sequence: Amino acid sequence (one letter code) of the isotopologue species including the type of acetyl group of the acetyllysine residues; K(ac12C): 12C2H3 acetyl group, K(ac13C): 13C2H3 acetyl group, K(ac*): 13C2D3 acetyl group (calculated in section 4).

Note: Only the precursors isobaric at MS1 level have to be included for MS2 quantification (in case of the H3(18-26) isotopologue species: VII*/VIII* (m/z 536.8228), II*/III* (m/z 538.3322), and V/VI (m/z 539.3356)).

Table S6: Sample table for MS2 fragment ion quantification using PASTAQ for isobaric H3(18-26) isotopologue species.

sample_name	precursor_min_RT	precursor_max_RT	precursor_mz	precursor_sequence
T4_C_replicate_1	21.4	22.4	536.8228	K(ac12C)QLATK(ac13C)AAR
T4_C_replicate_1	21.4	22.4	538.3322	K(ac12C)QLATK(ac*)AAR
T4_C_replicate_1	21.4	22.4	539.3356	K(ac13C)QLATK(ac*)AAR

5.2 In addition, a fragment table (*.csv, Table S7) must be generated containing the following parameters for each precursor specified in the sample table (see 5.1):
precursor_mz: the theoretical m/z of the precursor of the isotopologue species (calculated in section 4).
fragment_ion: type of fragment ion (calculated in section 4)
fragment_sequence: Amino acid sequence (one letter code) of the fragment ion including the type of acetyl group of the acetyllysine residues; K(ac12C): 12C2H3 acetyl group, K(ac13C): 13C2H3 acetyl group, K(ac*): 13C2D3 acetyl group (calculated in section 4).
fragment_mz: Theoretical m/z of the fragment ion (calculated in section 4).

Table S7: Fragment table for MS2 fragment ion quantification using PASTAQ for isobaric H3(18-26) isotopologue species. Table includes the b-fragment ions analyzed for both isobaric precursors of the single-acetylated H3(18-26) peptide (species II/III (m/z 538.3322), and species V/VI (m/z 539.3356)).

precursor_mz	fragment_ion	fragment_sequence	fragment_mz
539.3356	b1	K(ac13C)	173.1196
539.3356	b2	K(ac13C)Q	301.1782
539.3356	b3	K(ac13C)QL	414.2622
539.3356	b4	K(ac13C)QLA	485.2993
539.3356	b5	K(ac13C)QLAT	586.3470
539.3356	b1	K(ac*)	176.1384
539.3356	b2	K(ac*)Q	304.1970
539.3356	b3	K(ac*)QL	417.2810
539.3356	b4	K(ac*)QLA	488.3181
539.3356	b5	K(ac*)QLAT	589.3658
539.3356	y4	K(ac*)AAR	492.3243
539.3356	y5	TK(ac*)AAR	593.3720
539.3356	y6	ATK(ac*)AAR	664.4091
539.3356	y7	LATK(ac*)AAR	777.4931
539.3356	y8	QLATK(ac*)AAR	905.5517
539.3356	y4	K(ac13C)AAR	489.3055
539.3356	y5	TK(ac13C)AAR	590.3532
539.3356	y6	ATK(ac13C)AAR	661.3903
539.3356	y7	LATK(ac13C)AAR	774.4743
539.3356	y8	QLATK(ac13C)AAR	902.5329

5.3 Run the MS2 analysis script.
5.4 Select the sample table (see 5.1) and fragment table (see 5.2).
5.5 Set the resolution (MS1 and MS2 level) and input/output directories. If desired, plots can be generated for each fitted MS2 peak and precursor scan by toggling the Generate plots checkbox (Figure S15).
5.6 Start the analysis to extract the MS2 spectra within the precursor m/z and retention time tolerance window as given in the sample table (see 5.1) (Note: It is not necessary to specify a tolerance range for m/z, as the width of peaks in the m/z dimension only depends on the resolution and type of the MS analyzer). Each MS/MS spectrum is searched for the theoretical fragments of interest obtained from the fragment table (see 5.2) by extracting a slice of data centered around the theoretical m/z. Finally, the fragments are quantified by performing a weighted Gaussian curve fitting using non-linear least squares. If the fitting is successful, the area under the curve is calculated using the trapezoidal rule and this number is saved, along with the raw total intensity, fitted height, fitted m/z, and fitted width of the peak (sigma m/z) (Table S8). To ensure the fitting is correct, a plot for each raw data slice and its corresponding Gaussian fitting model is generated (Figure S16).
5.7 Export the results as *.csv.

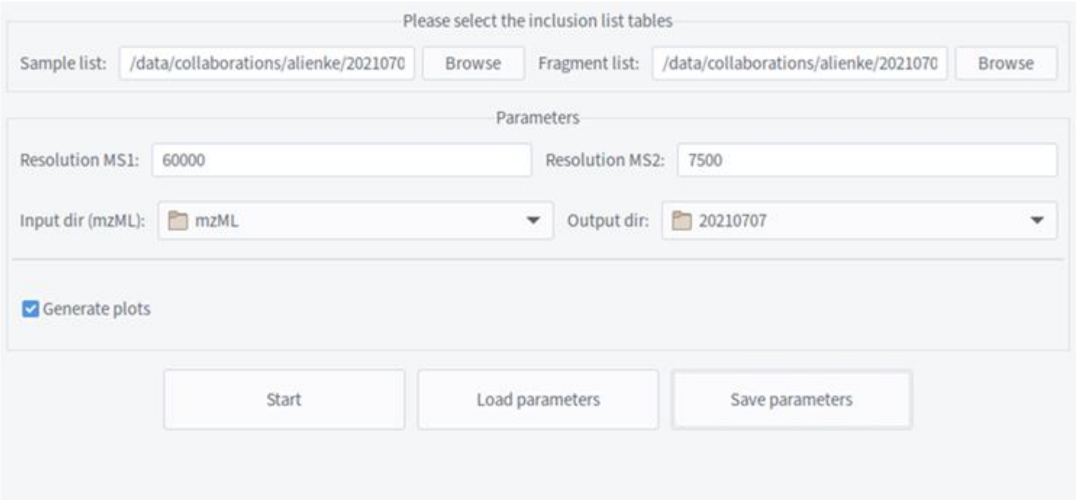

Figure S15: Graphical user interface (GUI) of PASTAQ

Table S8: Fragment ion quantification results presented in the PASTAQ MS2 output file, exemplified for H3(18-26) isotopologue species V*/VI* (m/z 539.3356).

precursor_mz	fragment_ion	fragment_sequence	fragment_mz	raw_height	raw_total_intensity	fitted_height	fitted_mz	fitted_sigma_mz	fitted_area	fitted_r2
539.3356	b1	K(ac13C)	173.12							
539.3356	b2	K(ac13C)Q	301.18	209405.77	860433.26	217209.28	301.1793	0.0173	856612.47	0.9985
539.3356	b3	K(ac13C)QL	414.26	87715.75	339880.08	86332.16	414.2591	0.0283	336515.19	0.9975
539.3356	b4	K(ac13C)QLA	485.3	15897.94	68177.57	16416.25	485.3002	0.0424	61771.27	0.9992
539.3356	b5	K(ac13C)QLAT	586.35							
539.3356	b1	K(ac*)	176.14							
539.3356	b2	K(ac*)Q	304.2	1510762.00	6304633.45	1586773.79	304.1986	0.0176	6264575.56	0.9981
539.3356	b3	K(ac*)QL	417.28	472815.56	1863599.71	470321.31	417.2829	0.0285	1855881.42	0.9984
539.3356	b4	K(ac*)QLA	488.32	108379.17	412028.12	106193.61	488.3108	0.0358	408451.00	0.9975
539.3356	b5	K(ac*)QLAT	589.37	210431.06	855868.36	217334.42	589.3521	0.0477	850817.23	0.9987
539.3356	y4	K(ac*)AAR	492.32	42535.59	158639.18	42105.31	492.3237	0.0355	155931.30	0.9975
539.3356	y5	TK(ac*)AAR	593.37	143744.95	564838.13	145381.50	593.3733	0.0477	562182.28	0.9987
539.3356	y6	ATK(ac*)AAR	664.41	274766.19	1074440.84	272807.18	664.4106	0.0568	1069125.98	0.9981
539.3356	y7	LATK(ac*)AAR	777.49	477557.63	1861356.96	471998.68	777.4939	0.0721	1853348.99	0.9981
539.3356	y8	QLATK(ac*)AAR	905.55	70055.13	274602.42	70747.64	905.5509	0.0899	271650.80	0.9984
539.3356	y4	K(ac13C)AAR	489.31	270827.88	1042110.50	265326.79	489.3079	0.0361	1039632.99	0.9977
539.3356	y5	TK(ac13C)AAR	590.35	865495.56	3347937.42	848685.74	590.3554	0.0478	3336221.99	0.9980
539.3356	y6	ATK(ac13C)AAR	661.39	1805236.63	7376815.45	1856871.97	661.3920	0.0566	7334426.40	0.9982
539.3356	y7	LATK(ac13C)AAR	774.47	3421384.25	13340209.79	3368204.46	774.4753	0.0719	13279589.38	0.9979
539.3356	y8	QLATK(ac13C)AAR	902.53	584597.19	2391379.01	600004.82	902.5321	0.0901	2378860.29	0.9979

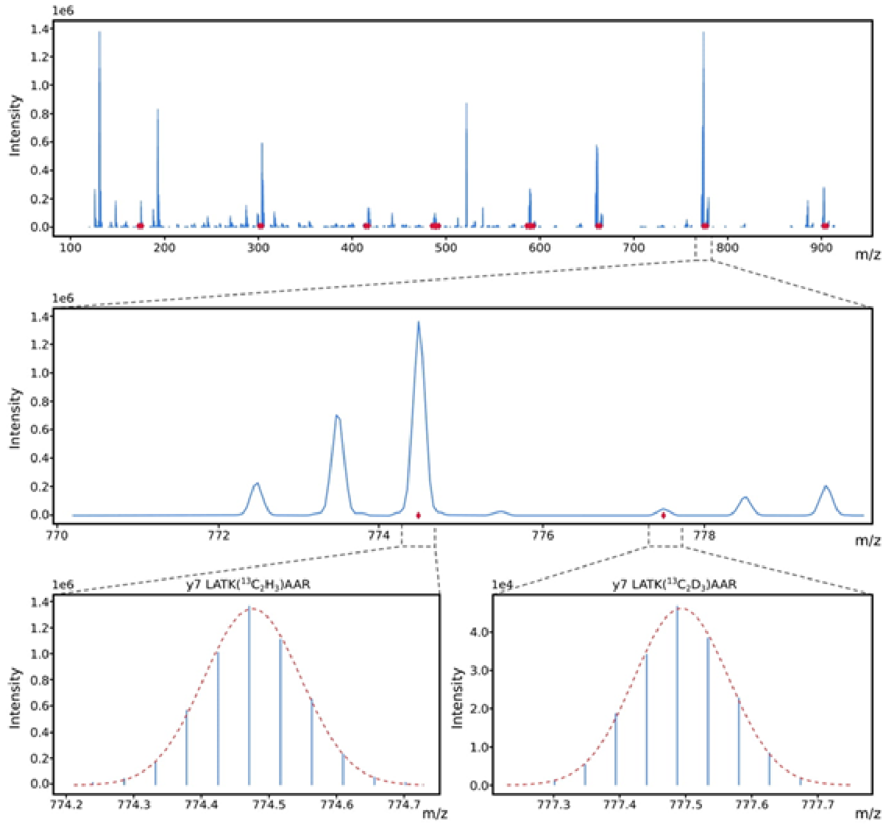

Figure S16: Gaussian fitting of detected y7-ion fragments of precursor isotopologue species V*/VI* (m/z 539.3356). (Top) Precursors MS/MS spectra within the sample table (5.1). The points of interest specified in the fragment table (red diamonds) are used for extraction of targeted fragment ions. (Middle) Magnification of two target ions of different intensities. (Bottom) Raw sampling points (blue stems) and the fitted Gaussian peak (dashed red line) for targeted ions.

6. Python: read in and calculate site-specific quantification

For the site-specific quantification of isobaric isotoplogue species, a relative quantification of the isobaric species has to be performed at the MS2-level. The relative abundance of the isobaric isotopologue species is multiplied with the corresponding MS1 abundance. For this, we use the scripts MS2_abundances_H3.py and Site-specific_intensities_H3.py, which can be downloaded at https://github.com/functional-proteomics-and-metabolomics/CoMetChem.

6.1 For relative quantification of the site-specific isotopologue species at MS2 level run the Python script MS2_abundances_H3.py using the MS2 data from section 5.6 as an input. Specify the PATH were the PASTAQ outputs are stored and the dictionaries REPLICATES/TIMEPOINTS.

6.2 The script Site-specific_intensities_H3 takes the Results.csv file from the script MS2_abundances_H3.py and the file containing the MS1 data (see 2.3) as input and corrects the results for the MS1 abundance. The SPECIES dictionary is necessary and has to be updated for the analysis of different isotopologue species.

6.3 The *.csv output file is used for further analysis

7. Calculation of site-specific half-lives and turnovers

7.1 Read in the file with the site-specific intensities into a python interpreter of your choice.

7.2 Using the SciPy package, fit an exponential curve over the ratio of heavy to heavy + light isotopologues over time using the following equation:

$$\frac{H}{H+L} = p \cdot \left(-e^{-kt} + 1\right)$$

The exponential factor k (turnover rate) can then be used to calculate the half-life of a species as

$$t_{1/2} = \frac{-ln(0.5)}{k}$$

Figure S17 shows the corresponding for the single-acetylated K18ac H3(18-26) species.

7.3 To calculate abundance-corrected turnovers, the turnover rate (k) has to be multiplied with the abundance of the species (A).

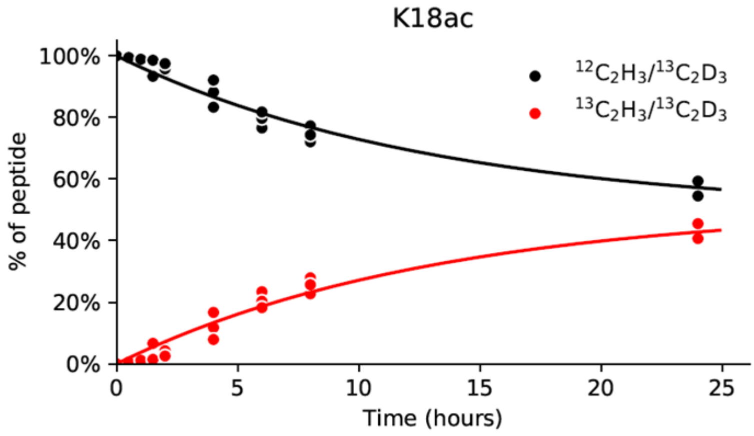

Figure S17: Site-specific label incorporation and label loss of the single-acetylated H3(18-26) species with an acetyllysine at K18. The black and the red line show the fit for an exponential decay or growth.

8. Calculation of site-specific reaction rates of acetylation and deacetylation

8.1 To calculate the site-specific acetylation and deacetylation rates, a linear fit was performed over the abundance of different species in the first 8 hours using the python-based ecosystem SciPy and the function linregress (Figure S18). The slope dx/dt and the intercept (Ax, abundance at T=0) are needed for further calculations.

8.2 Acetylation and deacetylation rates can be determined for the reactions outlined in Figure 3 in the main manuscript using the following equations:

$$\frac{dx}{dt} = -\upsilon_x \times A_x$$

In the case of deacetylation, where A_x is the abundance of the acetylated species x at T=0 and

$$\frac{dx}{dt} = \upsilon_x \times A_{substrate}$$

In the case of acetylation, where $A_{substrate}$ is the abundance of the non-acetylated species at T=0.

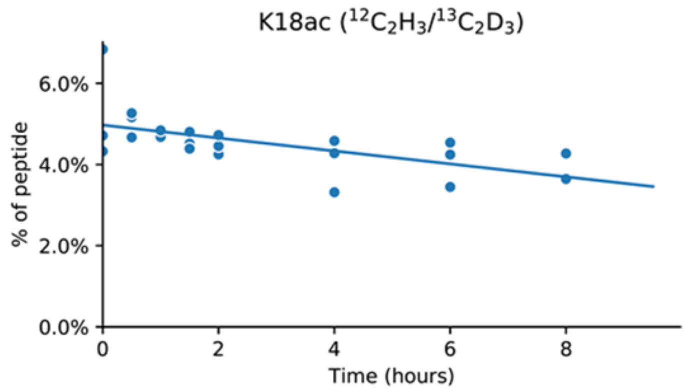

Figure S18: Linear fit over the abundances of the singly-12C acetylated species in the first 8 hours of the time course.

Note: The data processing pipeline is currently operating in a targeted way. The extracted ion chromatograms of the isotopes of the different isotopologue species must be generated in FreeStyle (section 1) or a similar program that allows to quantify MS1 isotopes and provide EICs of the isotopes of the isotopologues as *.csv. In addition, the sequence and modification of the isobaric isotopologues species must be specified in the Python script to calculate isotopologue-specific fragment ions. The data processing modules FreeStyle (section 1) and Fragmentation.py (section 4) can be easily adjusted to analyze any isotopologue species of interest. For a more global analysis approach, the Fragmentation.py module can be linked to the output of database search engines if they specify peptide sequences in the single-letter amino acid code including the specification of the modified acetyllysine as described in Section 4, and the FreeStyle module can be exchanged by a program that allows to quantify MS1 isotopes and provide EICs of the isotopes of the isotopologue species as *.csv.

References

[1] A. S. Brotons, J. Eriksson, M. Kwiatkowski, J. Wolters, I. Kema, A. Barcaru, F. Kuipers, S. Bakker, R. Bischoff, F. Suits, P. Horvatovich, *available at Research Square* **2021**.

[2] C. Feller, I. Forne, A. Imhof, P. B. Becker, *Mol Cell* **2015**, *57*, 559-571.

[3] J. Dietze, A. v. Pijkeren, M. Ziegler, M. Kwiatkowski, I. Heiland, *bioRxiv* **2020**.
